# Role of nanoclusters in the design and development of novel strategies for diagnosis and treatment of infectious diseases

**DOI:** 10.1039/d6na00472e

**Published:** 2026-09-01

**Authors:** Chinekwu Sherridan Nwagwu, Onyinyechi Lydia Nwachukwu, Stephen Chijioke Emencheta, Adaeze Linda Onugwu, Ezinwanne Nneoma Ezeibe, Chinenye Nnenna Ugwu, Petra Obioma Nnamani, Clemence Tarirai, Kenneth Ofokansi, Anthony Attama

**Affiliations:** a Drug Delivery and Nanomedicines Research Laboratory, Department of Pharmaceutics, Faculty of Pharmaceutical Sciences, University of Nigeria Nsukka Nigeria chinekwu.nwobi@unn.edu.ng adaeze.onugwu@unn.edu.ng; b Department of Pharmaceutical Technology and Industrial Pharmacy, University of Nigeria Nsukka Nigeria; c Department of Pharmaceutical Microbiology and Biotechnology, University of Nigeria Nsukka Nigeria; d Centre for Research Impact and Outcome, Chitkara College of Pharmacy, Chitkara University Rajpura 140401 Punjab India; e Department of Pharmaceutical Sciences, Faculty of Sciences, Tshwane University of Technology Pretoria South Africa; f Division of Microbiology and Infection, University of Leicester Leicester LE1 7RH UK

## Abstract

Despite major advances in sanitation, vaccination, and antimicrobial chemotherapy, infectious diseases continue to pose a huge threat to global health. The ever-increasing occurrences of antimicrobial resistance, emerging pathogens, and persistent diagnostic delays further exacerbate this situation. Over the years, nanotechnology has generated a lot of material platforms to address these failures, and in this study, we critically examine nanoclusters-ultrasmall, atomically precise assemblies (<2 nm cores) with discrete electronic states, as an under-recognized but potentially transformative class of materials for infectious disease diagnostics and therapeutics. Their quantized optical properties, precision ligand chemistry, and unusually high surface-to-volume ratios enable behaviours fundamentally inaccessible to larger nanomaterials, including ratiometric fluorescence reporting, rapid redox-driven antimicrobial activity, and programmable bio-recognition at near-molecular resolution. First, we disentangle NCs from larger nanomaterials by focusing on the consequences of discrete electronic states, ultrasmall hydrodynamic radii, and well-defined ligand shells for transport, clearance, and interactions with pathogens and host-derived biomolecules. Furthermore, the review synthesizes advances in NC-enabled diagnostics, including DNA and aptamer templated Au, Ag, and Cu NC probes, CRISPR/Cas integrated ratiometric sensors, and nanozyme-based readouts that routinely achieve single colony-forming unit or sub-femtomolar detection of bacterial, viral, fungal, and protozoan nucleic acids and antigens. On the therapeutic side, we discuss how the same structural characteristics can be exploited to engineer NCs that mediate controlled ROS generation, membrane and biofilm disruption, quorum sensing interference, photothermal and NIR II photodynamic therapy, and ligand-directed drug delivery, with case studies spanning multidrug-resistant bacteria, opportunistic fungi, and selected parasitic infections. At the same time, we looked at some of the major barriers to clinical translation that remain unaddressed. We show with this study that nanoclusters are not merely “smaller nanoparticles” but a distinct chemical system, capable of enabling modular, multiplexed, and clinically relevant infectious disease interventions. To realize this potential, future work must couple atomic-level design with rigorous pharmacokinetic, toxicodynamic, and translational engineering frameworks.

## Abstract 2

The unique physicochemical properties of nanoclusters, including ultrasmall size, tunable surface chemistry, high surface-to-volume ratio, and distinctive optical behavior, enable their use in the diagnosis and treatment of bacterial, viral, fungal, and parasitic infections.

## Graphical abstract

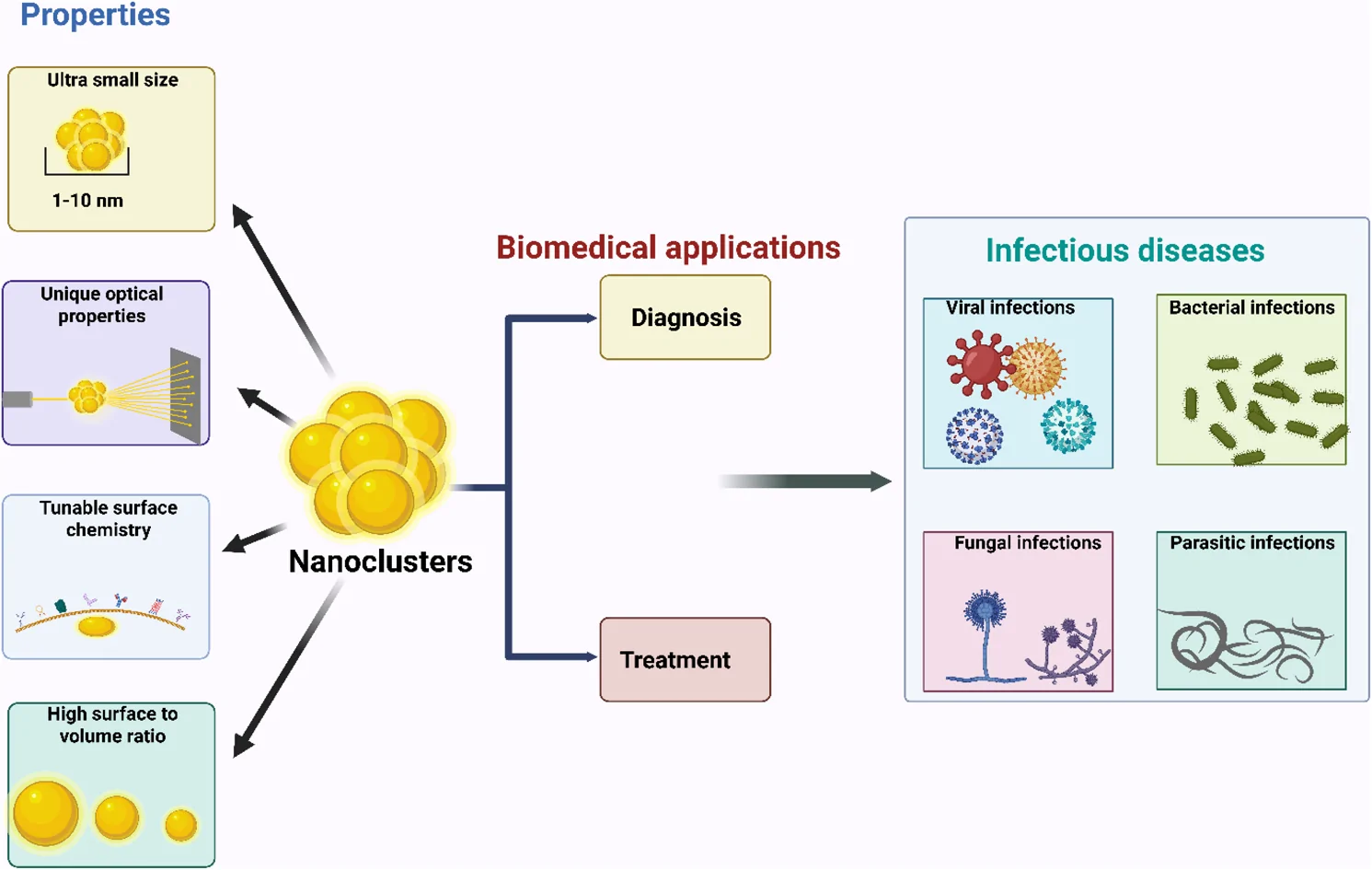

## 1 Introduction

Infectious diseases refer to illnesses that are caused by infectious pathogens, such as bacteria, viruses, fungi, and parasites, and can be transmitted from one person to another. These diseases represent major challenges to global public health and are responsible for millions of mortalities each year, most of which occur in developing countries.^1–5^ Bacteria such as *Enterococcus faecium*, *Staphylococcus aureus*, *Klebsiella pneumoniae*, *Acinetobacter baumannii*, *Pseudomonas aeruginosa*, and *Enterobacter* spp. are major pathogens responsible for approximately one-third of infectious deaths.^1–5^ Viruses have also been reported to cause extensive harm to human health across the globe, with infections such as novel influenza A (H1N1) flu, Ebola, hemorrhagic fever, Middle East respiratory syndrome (MERS), and Zika virus disease (ZVD) and the more recent pandemic caused by severe acute respiratory syndrome (SARS-CoV-2), claiming millions of lives.^1–5^ Furthermore, fungi such as those in the Mucormycetes class have been reported to cause various illnesses that affect the lungs and sinuses, usually as a result of inhalation of their spores, while many other fungi have been implicated in infections such as ringworms, jock itch, as well as onychomycosis.^1–5^ Parasites such as *Giardia lamblia*, which causes beaver fever, and *Entamoeba histolytica*, responsible for amoebic dysentery, as well as a variety of intestinal worms, cause debilitating illnesses in humans.^1–5^ Although there have been major advances in sanitation, vaccination, and antibiotic treatment, which have greatly reduced their impact, infectious diseases still cause a huge proportion of illness and death worldwide.^1–5^ Population growth, the rising problem of antibiotic resistance, and the continual appearance of new pathogenic organisms are major contributors to the challenges of infectious diseases, thereby increasing the burden on public health systems.^4,5^ Various reports also show that both long-standing and newly emerging infections remain major contributors to illness and mortality, especially in low- and middle-income countries.^6–8^ As such, researchers are looking for new diagnostic and treatment approaches that are both fast and accurate in order to tackle the challenges of existing and emerging pathogens.

Although conventional diagnostic tools and therapies have played a huge role in the control of infectious diseases, their performance is often limited by various challenges. For instance, diagnosis using culture-based methods is still widely used because they can definitively identify pathogens, but these processes take time and depend on the pathogen's viability as well as the ability to be cultured in the lab.^4,9,10^ Moreso, molecular methods such as PCR can detect infections more quickly and with high specificity, but they often focus on a limited set of targets, can be affected by contamination, and may miss infections under certain conditions, leading to false-negative results.^4^ On the other hand, while serological tests and relying on clinical symptoms expand the diagnostic options, these methods are also faced with challenges regarding sensitivity, specificity, and timely detection, problems that become even more serious, especially in the diagnosis and treatment of infections caused by emerging pathogens.^4^ The process of treating these infections also faces several challenges. Despite the fact that antibiotics, antivirals, and antifungals have saved countless lives, challenges such as antimicrobial resistance, off-target effects, and delivery inefficiencies are increasingly undermining their effectiveness.^5^ These challenges have brought about the need for new and better strategies to tackle infectious diseases.

For over 20 years, nanotechnology has provided important tools for addressing various challenges in managing infectious diseases. Its versatile applications have brought about the development of very sensitive diagnostic tools, as well as targeted drug delivery systems that help to reduce off-target effects and, more recently, theranostic agents that combine diagnosis and therapy into a single system.^2,11^ Many nanomaterials have also been designed to cross biological barriers, improve pharmacokinetics as well as modulate interactions between the host and pathogens. As a result, these advances have helped to improve treatment effectiveness while reducing toxicity.^12–14^ One of such systems is nanoclusters, a unique class of ultrasmall materials comprising only a few to several hundred atoms. Nanoclusters have also been developed for the diagnosis and treatment of infectious diseases.^15,16^ These materials possess distinct physicochemical properties not observed in larger nanoparticles. Their structures, tunable surface chemistry, and size-dependent electronic and optical characteristics provide features for precise pathogen detection and more effective therapeutic delivery.^17,18^

Many studies have explored the various uses of nanoclusters in biomedicine; however, existing reviews have only partly covered their applications in the diagnosis and management of infectious diseases. For instance, there are reports that have mainly focused on multifunctional ultra-small metal nanoclusters and bacterial infections. These studies discuss in detail the antimicrobial mechanisms of metal nanoclusters, but offer limited insight into the use of these systems for other pathogens apart from bacteria.^10,17^ Other reviews that focused on gold nanoclusters for bacterial detection and therapy have provided detailed accounts of photoluminescent sensing and antibacterial action, yet have not considered other metals or pathogen classes.^2,5^ Much broader nanocluster reviews have often been centered on oncology, where targeting strategies and therapeutic applications are tailored to tumor biology rather than infectious disease pathophysiology.^19^ Furthermore, other nanoparticle-focused articles have surveyed gold and silver systems in various biomedical contexts, but without distinguishing nanoclusters from larger particles or examining their unique properties.^2,20–22^

In contrast, this review aims to highlight the application of nanoclusters across bacterial, viral, fungal, and parasitic infections, examining how atomic composition, ligand chemistry, and physicochemical properties contribute to both diagnostic and therapeutic efficacy. We not only compare nanoclusters with conventional diagnostic and therapeutic approaches, as well as with larger nanoparticles, but we also examine the key factors that continue to challenge their clinical translation. In addition, we highlight possible strategies for overcoming these barriers in the context of infectious diseases. In summary, the study aims to move the discussion beyond laboratory demonstrations and position nanoclusters as applicable tools for next-generation diagnosis and treatment of infectious diseases.

## 2 Overview, characteristics, and methods of preparation of nanoclusters

Nanoclusters (NCs) are mesoscopic particles of a few to thousands of atoms. They are nanostructures with sizes between 1 and 10 nm. Their cluster core is usually less than 2 nm; however, nanoclusters are usually stabilized with protective groups/ligands that increase their dimensions.^23^ Structurally, NCs consist of noble or transition metal core (*e.g.*, gold, silver, platinum, copper) surrounded by a protective ligand shell (such as thiolates, phosphines, halides and alkynyls). These protective ligands have a great impact on their configuration, chirality, luminescence, stability and activity.^24–26^ NCs are represented chemically as [X_*a*_Y_*b*_]^*c*^, where *a*, *b*, and *c* are the number of metals (X), ligands (Y), and charges on the NC, respectively.

The terms nanoparticles and nanoclusters are usually confused or used interchangeably. However, nanoparticles are larger particles of nanoscale dimension, with sizes ranging from 10–100 nm. Nonetheless, the emphasis on the distinction between nanoparticles and nanoclusters focuses on the unique advantages of the latter, which often depend strongly on their atomic structure, ligand environment, and specific application scenarios. Unlike nanoparticles, NCs do not show a surface plasmon resonance (SPR) effect; instead, they exhibit unique electrochemical, optical and catalytic properties due to their ultrasmall size (Fig. 1). Nanoclusters are, therefore, known as an intermediate structure between atoms and nanoparticles.^27^ At nanocluster size, comparable to the size of Fermi electrons, there is a strong quantum effect of electrons, which causes the breakdown of the continuous energy levels into discrete energy bands (Fig. 1). Unlike nanoparticles, it is easy to design nanoclusters with specific and uniform morphological features. X-ray crystallography and mass spectroscopy hyphenated techniques can be used to unravel their precise atomic structure, including the exact size and charge of the metal core and the number of protective ligands. In contrast, nanoparticles have variable properties, dramatically limiting their reproducibility and clinical translation.

**Fig. 1:**
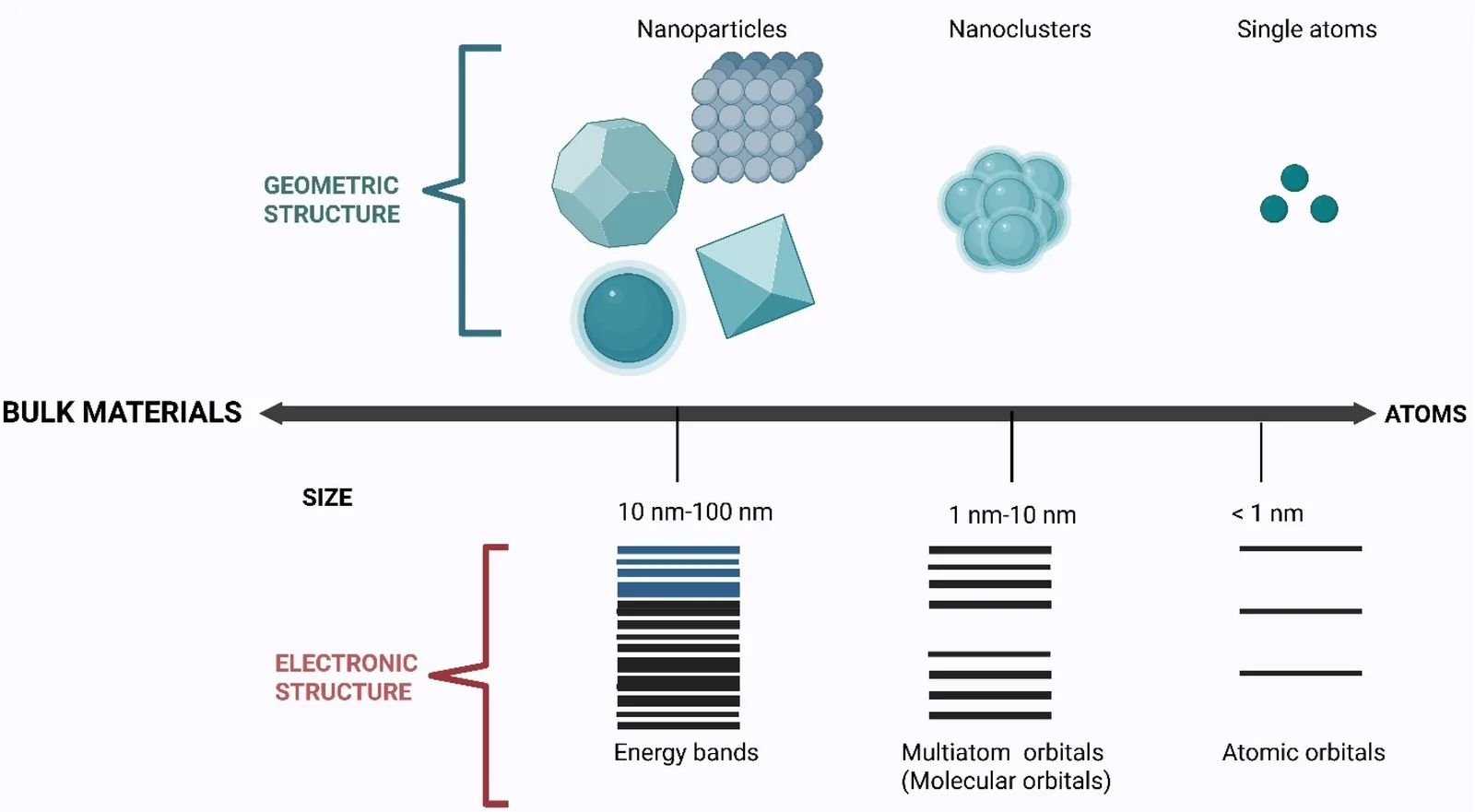
Size-dependent structural and electronic evolution from bulk materials to single atoms. Schematic illustration showing the transition from bulk materials to nanoparticles (10–100 nm), nanoclusters (1–10 nm), and ultimately single atoms (<1 nm). As size decreases, geometric structures evolve from bulk-like crystalline arrangements to discrete cluster morphologies and isolated atomic species. Concurrently, the electronic structure transitions from continuous energy bands in nanoparticles to discrete multi-atom (molecular-like) orbitals in nanoclusters, and finally to well-defined atomic orbitals at the single-atom limit. This size-dependent evolution underpins the distinct physicochemical, optical, and catalytic properties observed across the nanoscale. (Image adapted from Liu and Corma (2018)^28^ Copyright© 2018 American Chemical Society. Image was created with the assistance of Biorender.

Characterization of metal NCs differs from that of conventional nanoparticles because their exact molecular composition and atomic arrangement must be established. Many studies use electrospray ionization mass spectrometry to evaluate the molecular mass, charge state, metal-to-ligand composition, as well as isotope distribution of a nanocluster.^29–31^ Tandem mass spectrometry can also be used to distinguish closely related cluster species and provide information on fragmentation pathways as well as reaction intermediates.^31^ Single-crystal X-ray diffraction provides the complete atomic structure, including the metal kernel, surface motifs, ligand coordination, bond distances, and positions of dopant atoms. It is therefore the most direct structural method when suitable single crystals can be obtained.^32^ Furthermore, conventional methods for nanoparticle characterization, such as transmission electron microscopy, dynamic light scattering, and powder X-ray diffraction, are equally useful for measuring size, morphology, and crystallinity of nanoclusters.^29,30,33^ However, these methods cannot independently confirm atomic precision. Some studies have employed other complementary methods such as X-ray photoelectron spectroscopy and X-ray absorption spectroscopy for elemental composition and oxidation state, nuclear magnetic resonance and infrared spectroscopy for ligand coordination, and UV-visible and photoluminescence spectroscopy for electronic and optical properties.^32^ Many studies use a combination of these techniques to confirm the composition, structure, and properties of metal nanoclusters.

Recent studies have shown that the properties of metal NCs can be controlled through precise changes in the metal core and ligand shell. Shi *et al.* reported an atomically precise Au_16_Cu_6_ NC with a near-infrared photoluminescence quantum yield above 99% in deaerated solution at room temperature. This finding showed that control of atomic composition and structure can greatly improve emission.^34^ He *et al.* compared two Au_18_(SR)_14_ NCs with the same number of gold atoms but different thiolate ligands. Interactions between the aromatic ligands promoted intersystem crossing and improved the stability of the NC, showing that the ligand shell can control excited-state behaviour.^35^ Dai *et al.* prepared Au_8_Ag_4_, Au_7_Ag_5_, and Au_6_Ag_6_ alloy NCs and found that the Au/Ag ratio changed their electronic structure and ROS generation. Au_6_Ag_6_ showed the strongest photocatalytic activity and achieved 99.9% antibacterial efficacy against Gram-positive and Gram-negative bacteria under light irradiation.^36^ These studies show that atomic control of the metal core and ligand shell can be used to adjust the optical, catalytic, and biomedical properties of metal NCs.

Due to their intriguing optical and electronic properties, nanoclusters have applications in various fields as catalysts, bio-imaging probes, drug delivery systems, and chemical and biological sensors.^37–42^ Nanoclusters possess desirable properties for diagnostic and therapeutic purposes. Such properties are small size, large surface area, adjustable surface chemistry as well as unique optical, electromagnetic, and catalytic features. For instance, gold nanoparticles are inactive against bacteria, but once the dimension is reduced to nanocluster size (≤2 nm), it becomes a potent antibacterial agent.^43^ The molecular-like characteristics of metal NCs impact the antimicrobial mechanism of nanoclusters. Silver and copper nanoclusters are excellent antibacterial agents due to the intrinsic antibacterial activity of the metals; however, gold nanoclusters have more stability and a better biocompatibility profile.^43^

NCs have attractive catalytic properties resulting from their unique geometric and electronic structure. The atomic packing structure is distinctively different from that of bulk materials, with a large surface area and a surface rich in low-coordination atoms. Also, the quantized energy levels in nanoclusters aid in activating reactants as the electrons are excited between different energy levels. Furthermore, their readily modifiable structural properties are of great benefit in catalysis.^44^ The emergence of nanoclusters has paved the way for new catalytic processes. An essential property of nanoclusters utilized in optical biosensing is aggregation-induced emission (AIE). When nanoclusters assemble, an enhanced emission intensity superior to that of individual clusters is obtained. Studies have shown that the intensity of this emission is influenced by solvent properties, nanocluster concentration, temperature, pressure, and the presence of ligands or biomolecules.^43^ These factors can be modified to regulate the size and morphology of self-assembled nanostructures and their biosensing activity.

Metallic NCs are synthesized using two major methods: bottom-up or top-down methods. Other synthetic methods are ligand exchange and template-assisted methods. Bottom-up methods can be achieved *via* chemical, electrical, biological, or photo reduction, depending on the reducing agents/environment. The chemical method involves the reduction of metal ions to metal atoms in the presence of a reducing or stabilizing agent. Common reducing/stabilizing agents are NaBH_4_, thiolates (for example, 6-mercaptohexanoic acid, glutathione), proteins and peptides (for example, albumin, lysozyme, transferrin, and hemoglobin), alkynyls (for instance, acetylides), DNAs, and polymers have been used.^45–50^ The reduction process is followed by gradual nucleation and growth on the ligand surface to get the desired nanocluster size. The bottom-up method is commonly utilized for synthesizing NCs because it is simple, fast, and efficient. The major hindrance is the control of the reaction kinetics, which affects the quality of the NCs. In a top-down chemical etching or size-focusing method, large nanoparticles are etched using ligands to produce small nanoclusters (Fig. 2). This method takes time and gives poor yields.^51^ However, the process can be improved by optimizing the etching conditions, such as the etching time, reaction temperature, and the ratio of etchant to metal precursors. Mild etching conditions are required for the efficient synthesis of nanoclusters. On the other hand, template-assisted methods involve the use of templates/ligands such as polymers, dendrimers, proteins, and peptides as scaffolds for synthesizing nanoclusters. The size and morphology of the nanocluster can be effectively monitored during the preparation. A ligand exchange method makes it possible to synthesize metal NCs through selective replacement of the ligand on the surface of the nanosystem. The method may involve complete or partial ligand exchange, with or without modifying the metal core (Fig. 2).

**Fig. 2:**
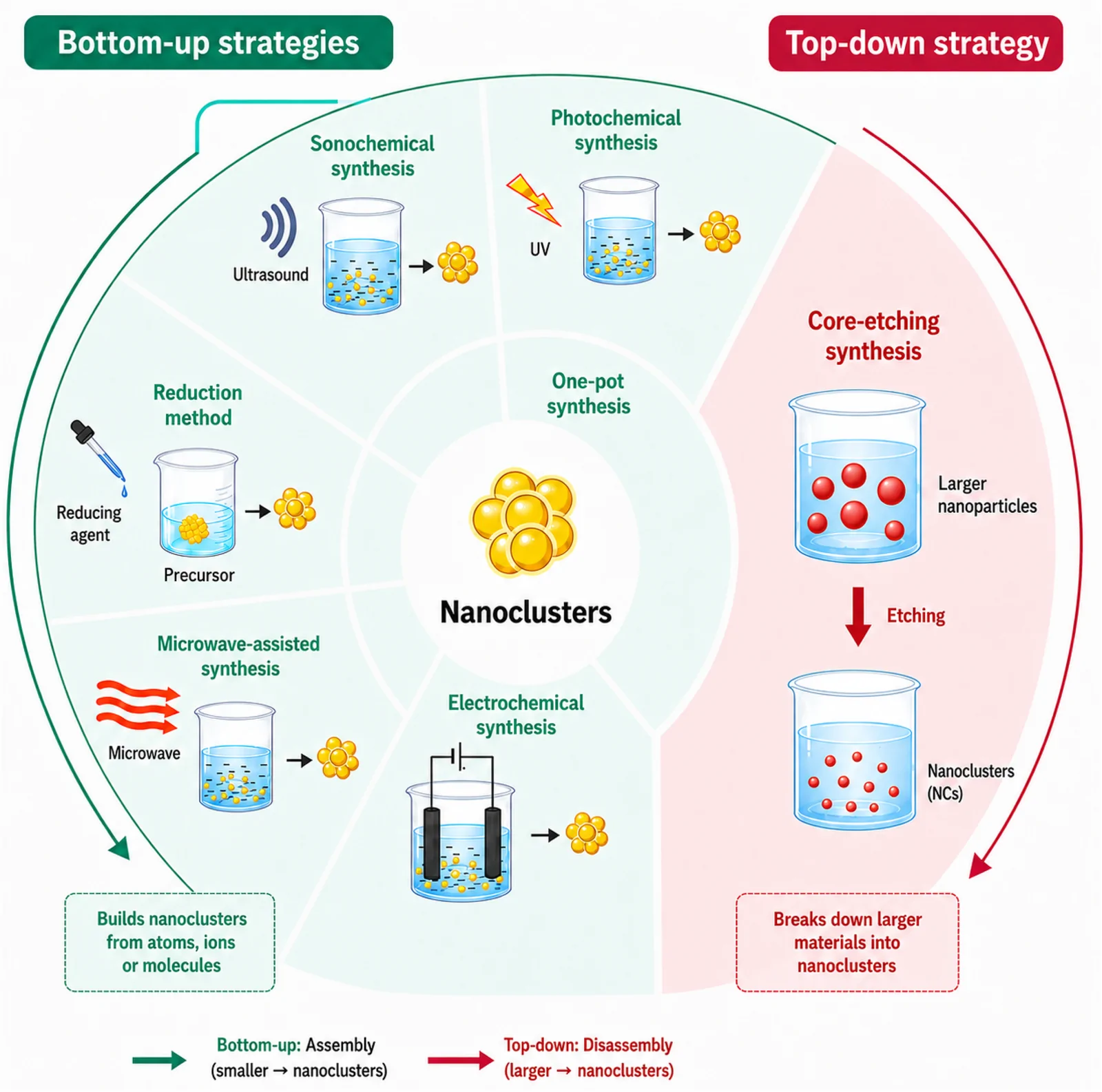
Overview of representative synthetic routes for metal nanoclusters (NCs). Illustrated methods include reduction-based synthesis, sonochemical, photochemical, microwave-assisted, electrochemical, and one-pot ligand-mediated approaches, as well as a core-etching strategy in which larger nanoparticles are transformed into ultrasmall, ligand-stabilized NCs. Image created with the assistance of Biorender.

The properties of NCs are greatly influenced by their size and composition; hence, it is crucial to produce NCs with precise dimensions and compositions for the intended applications. Precise tuning of the nanocluster size can be achieved by controlling the conditions for the synthetic process. The commonly used reducing agent, NaBH_4_, produces a fast reaction leading to polydisperse nanoclusters. The careful control of the reduction kinetics by modifying the concentration of the reducing agents, pH of the solution and the solvent can produce narrow-sized nanoclusters.^44^ For instance, the addition of NaOH decreased the reducing capacity of NaBH_4_ on Au ions and hence achieved the desired size of the nanoclusters.^52^ Another way of decreasing the rate of the reduction reaction is using mild reducing agents such as formic acid and dimethyl formamide. Compared with the chemical reduction strategy, other reduction-based methods use light, microwave, ultrasonication or biological systems to create a mild reduction condition and to provide clean and green procedures for nanocluster synthesis (Fig. 2). There is a growing interest in the use of biological systems with both reducing and protective activity as a more biocompatible and environmentally friendly route. For instance, it was reported that bovine serum albumin gold nanocluster was formed through the reduction of the gold ion by aromatic amino acid groups in the protein molecule.^53^

In addition to controlling the reduction rate, recent studies have provided a clearer understanding of how atomically precise metal NCs form. Their synthesis can be seen as a sequence of molecular reactions. These include metal–ligand complex formation, reduction, cluster growth, structural rearrangement, and then size focusing. The relative rates of these steps determine the final atomic composition as well as the structure. Electrospray ionization mass spectrometry, ESI-MS, is widely used to identify stable intermediates and to follow their conversion during synthesis.^30,54^

Yao *et al.* examined the seed-mediated growth of [Au_25_(SR)_18_]^−^ into [Au_44_(SR)_26_]^2−^ and identified 35 Au(i)-thiolate complexes and NC intermediates. The process involved the initial accumulation of Au_25_, seed-mediated cluster growth, and thermodynamically controlled size focusing. Both LaMer-like growth and aggregative growth contributed to the size evolution. The reactions were driven by sequential two-electron increases in the valence electron count.^55^ In another study, the conversion of [Au_23_(SR)_16_]^−^ into [Au_25_(SR)_18_]^−^ occurred through the exchange of Au-thiolate surface motifs and a symmetry-breaking rearrangement of the metal core. The authors also employed tandem mass spectrometry, which provided information on bond stability and the different structural changes that occurred during this conversion.^54,55^ Reaction temperature can determine whether a kinetic or thermodynamic product is formed. Wang *et al.* obtained a metastable Ag_54_ NC at 20 °C and a more thermodynamically stable Ag_33_ NC at 80 °C from the same starting materials. The structures were directed by internal CrO_4_^2−^ and/or Cl^−^ templates. This study shows that temperature can alter the formation pathway and permit the selective isolation of a defined NC.^56^ Precursor identity and counterions can also control atomic composition. In a series of thiolate-protected Au–Cu NCs, the Au precursor and counter cation controlled the formation of [Au_12+*n*_Cu_32_(SR)_30+*n*_]^4−^ clusters, where *n* was 0, 2, 4, or 6.^30,36,57^ These findings support a molecule-like approach to NC synthesis, in which individual reaction steps are controlled to obtain a defined metal core, metal–ligand interface, and ligand shell.^30,36,55^

Many strategies have been employed to enhance nanoclusters' stability, including the use of protective ligands and alloying. Organic ligands such as thiolates, phosphines and alkynyls are usually used to cap the surface of the nanocluster to prevent aggregation and coalescing into larger particles.^58^ They also reduce the reactivity of the nanocluster by binding to unsaturated bonds. Thiol groups are widely explored as ligands for nanoclusters due to the formation of strong covalent bonds between the sulfur atoms in the ligands and metal atoms. Attachment of ligands to the surface of the metal atoms affects their optical, electronic and biological properties as well as enhances their stability. Stabilizing nanoclusters using ligands is based on the excellent interaction of a soft base with a soft acid. Soft bases such as thiolates, selenides and phosphines strongly interact with soft acids such as silver, gold, and copper nanoclusters.^59^ Notwithstanding, hard bases such as nitrogen and oxygen atoms can also be employed to synthesize NCs. Proteins are the trending biomolecules for stabilizing nanoclusters. Protein-stabilized nanoclusters exhibit high quantum efficiency and are highly stable under ambient conditions, making them potential drug delivery systems, imaging and sensing materials. For instance, bovine serum albumin-stabilized gold nanoclusters were developed to detect breast cancer.^60^

Generally, metal alloy NCs, such as AuAg NCs and AuPt NCs, exhibit adjustable bioactivities and higher stability profiles compared with single-metal nanoclusters. However, the effect of alloying on antimicrobial performance depends on the metal composition and the resulting electronic structure. While alloying may decrease antimicrobial activity by increasing stability and reducing ROS generation,^61^ recent data has shown that precise regulation of the alloy composition can also produce the opposite effect. Dai *et al.* demonstrated that increasing the Ag content of atomically precise Au_12−*x*_Ag_*x*_ nanoclusters increased their ROS-generation rates from 0.41 µM min^−1^ for Au_8_Ag_4_ to 0.81 µM min^−1^ for Au_7_Ag_5_ and 1.06 µM min^−1^ for Au_6_Ag_6_, resulting in enhanced photocatalytic antibacterial activity under light irradiation.^36^

## 3 Nanoclusters for diagnosis and treatment of infectious diseases

### 3.1 Nanoclusters for bacterial infections

The use of nanoclusters is a promising, innovative, and strategic tool for addressing the increasing resistance of disease-causing bacteria to drugs. Aside from enhancing the targeted delivery of therapeutic payload, it leverages different mechanisms, including cell wall and membrane disruption by direct interaction and subsequent compromising of the envelope and leak out of the bacterial cell content, membrane-fusion-based distribution of antimicrobial agents, generation of reactive oxygen species, such as hydrogen peroxide and hydroxyl and superoxide radicals, inducing oxidative stress in bacterial cells and damaging cellular macromolecules (lipids, proteins, and DNA), and interactions with intracellular elements, to eliminate pathogenic bacteria, quorum sensing interference and biofilm disruption as seen in Fig. 3.^62–66^

**Fig. 3:**
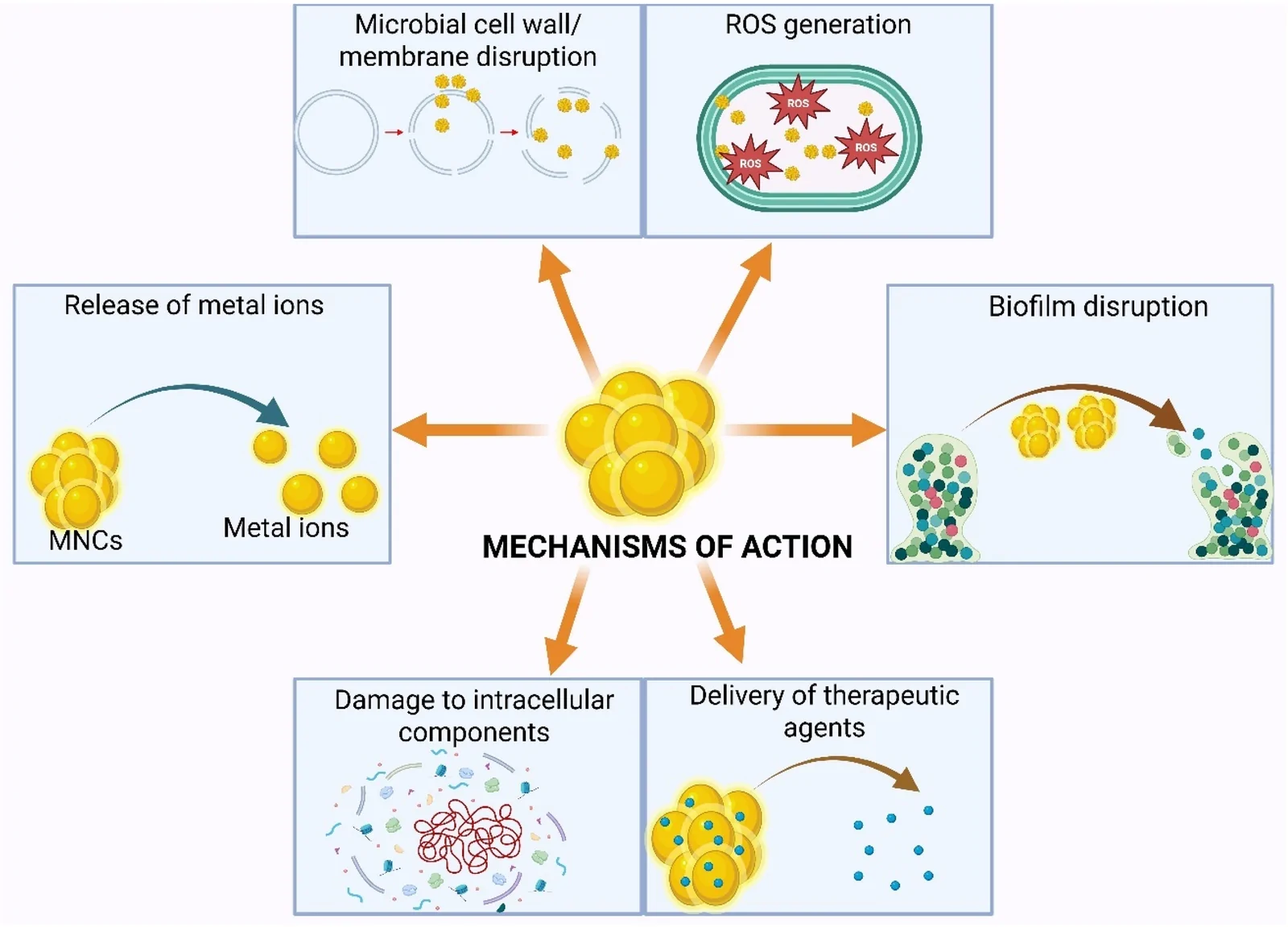
Mechanisms of action of metal nanoclusters (NCs). NCs exhibit antimicrobial activity *via* membrane disruption, ROS generation, metal ion release, biofilm disruption, intracellular damage, and delivery of therapeutic agents. Image created with the assistance of Biorender.

Recent advances in specific applications underscore the applicability of nanoclusters across bacterial diagnostics, phototherapy, drug delivery, and wound healing. For instance, Dan *et al.*^67^ investigated the enhancement of PDT for managing cancer and bacterial infections by using gold nanoclusters (BSA@Au) as near-infrared (NIR II) photosensitizers with catalase-like activity (catalyzing hydrogen peroxide to produce singlet oxygen). With traditional photosensitizers limited by high background fluorescence in the NIR-window and oxygen dependency, thus being hindered by hypoxic conditions for effective PDT, this approach aimed to utilize photosensitizers that can operate in the NIR-II window, offering apparent advantages, including improved signal-to-background ratios and reduced scattering. The BSA@Au, produced through the biomineralization method and characterized, possessed a core size of 2 nm (below renal threshold), exhibited strong NIR-II fluorescence, and demonstrated capacity for *in vivo* imaging due to its photostability and quantum yield, although its imaging ability was tested by visualizing tumours in 4T1 tumour-bearing mice. The nanocluster, in combination with hydrogen peroxide and laser irradiation, exhibited antibacterial activity by binding to methicillin-resistant *Staphylococcus aureus* (MRSA), resulting in bacterial killing and providing an opportunity for further elucidation of its mechanism of action.

On the diagnostic front, to enhance sensitivity and speed for detecting *E. coli*, Kotsifaki *et al.*^68^ developed a label-free, plasmon-enhanced optical sensing platform using an array of asymmetric split-ring gold plasmonic nano-aperture types, which support Fano-resonance mode matching the protein amide fingerprints. Designed with submicron gaps (∼0.41 µm), which facilitated bacterial trapping/localized field enhancement, employing off-resonance 530 nm laser excitation to minimize photodamage, and enabling Fano-enhanced Raman scattering. This resulted in the amplification of the weak Raman signals of the bacteria. The study demonstrated the potential of the nanostructure for ultrasensitive bacterial monitoring and detection, with the intensity of the Raman signal correlating with the bacteria's growth phases, peaking at the exponential phase and declining at the post-stationary phase.

In detecting *Salmonella typhimurium*, a known food pathogen, Ma *et al.*^69^ reported SCENT-Cas, an ultrasensitive detector integrating silver nanoclusters (AgNCs) and CRISPR/Cas12a technology, resulting in a radiometric fluorescent output. The authors utilized loop-mediated isothermal amplification (LAMP) to amplify the inVA gene, which is specific to the bacteria, and activated CRISPR/Cas12a for signal transduction, with the AgNCs integrated to enable dual-colour fluorescence output. The study demonstrated a linear correlation between the fluorescence intensity and the concentration of *S. typhimurium*, a sensitive limit of detection of 1 CFU mL^−1^, a selective and specific triggering of significant changes in the fluorescent ratio, and a promise of reliable detection of the bacteria in complex food matrices.

While Ma *et al.* synthesized silver nanoclusters by a low-temperature template-guided chemical reduction employing sodium borohydride as the reducing agent, Liu and his team^70^ developed hairpin DNA-stabilized copper nanoclusters (CuNCs) as a novel fluorescent biosensor for intracellular pathogens using ascorbic acid as the reducing agent in an enzyme-free room temperature solution phase system. These nanocomplexes were designed to detect *Chlamydia trachomatis*, an obligate intracellular bacterium that causes sexually transmitted chlamydia infections.^71^ This biosensor addresses the limitations associated with conventional detection methods, such as serological tests and polymerase chain reaction (PCR), particularly the issue of specificity and sensitivity. In the design of CuNCs, the hairpin DNA probe specifically recognises the DNA of *C. trachomatis* and the hairpin structure enables a conformational change upon hybridising with the target bacterial DNA, thereby producing a corresponding fluorescent output from the nanocluster. CuNCs exhibit fluorescence excitation and emission peaks at 345 and 606 nm, respectively, which are critical for detecting *C. trachomatis*. The probe then hybridizes with the omp1 gene of *C. trachomatis*, and the conformational change in the probe results in a quantifiable change in CuNC fluorescence. The biosensor demonstrates a linear response across various concentrations of the target DNA, has a low detection limit of 18.5 nM, as well as high selectivity against non-target microbial sequences. The study confirmed successful synthesis and target recognition using thorough characterisation *via* spectroscopy, gel electrophoresis, and TEM. Optimisation of probe design, reaction conditions, and buffer parameters further enhanced sensitivity. Validation in HeLa cell lysates yielded near-quantitative recovery, underscoring its potential for real-sample application. While this platform is not relatively novel, it offers a promising alternative for the early, accurate, and field-deployable diagnosis of *C. trachomatis* infections.

While Liu *et al.* explored the use of an intelligent DNA base-pairing (hairpin structures) template for sequence-specific targeting, Murugan *et al.*^72^ developed aromatic thiol-protected copper nanoclusters (CuNCs) *via* a straightforward one-pot synthesis method, focusing on the robust stability of the system through covalent metal–sulfur coordination, thereby protecting the copper core against oxidation and aggregation and enhancing their catalytic efficiency and their bioeffects, including bioimaging and antibacterial effects. The addition of a reducing agent to a mixture of aromatic thiol ligands and a copper salt solution resulted in nucleated copper growth. The hydrophobic sulfur ligands cap the surface, with resulting products having a particle size of 10 nm, having absorption peaks between 313 and 378 nm, indicating successful metal-to-ligand charge transfer. Among other activities assayed for (cytotoxicity and ability to degrade methylene blue), the CuNCs had antibacterial activities, specifically against *E. coli* and *S. aureus*, with a maximum inhibition zone diameter of 20 nm (at concentration of 100 µg) recorded against *E. coli*, and underlining the multifunctional capacities of the platform, but call for further studies, including the optimisation of the fluorescence emission for improved bioimaging capabilities.

The study by Liu *et al.*^73^ marks a major shift from individual, target-specific biosensing as seen in preceding reported studies to high-dimensional multi-analyte profiling. The authors developed a multichannel sensor array based on antimicrobial peptide-functionalized gold nanoclusters (AMP-AuNCs) and paired with machine learning algorithms. The nanocluster possessed the ability to bind distinctively and produced varying fluorescence patterns upon binding to different bacterial species. Specifically, the authors produced three different AMP-AuNCs using three different antimicrobial peptides with distinct hydrophobicity and charge. Optimisation studies found that the best reaction time between the AMP-AuNCs and the test bacteria is 30 minutes, with the pH being 9, which served as the best condition for distinguishing between the bacterial species. Against representative pathogenic bacterial species, including *Pseudomonas aeruginosa*, *E. coli*, *A. baumannii*, *Streptococcus epidermidis* and *S. aureus*, the AMP-AuNCs were evaluated for their bacterial identification performance by calculating the changes following interactions and generating heatmaps to visualize the distinct fluorescence response for each bacterium. Principal component analysis and hierarchical clustering demonstrated that the sensor array could effectively differentiate between bacterial species, achieving a high accuracy (100%) in classification. Furthermore, it recorded an 86.7% accuracy against clinical isolates of *E. coli* from urinary tract infections.

Similarly, Chang *et al.*^74^ introduced a papain-encapsulated platinum nanocluster (papain-Pt NCs), a dual fluorescent biosensor for selectively detecting and identifying lysozyme and Gram-positive bacteria, respectively. Made through a biomineralization process, papain-Pt NCs detected lysozyme at a limit of 16.94 nM, indicating the nanocluster as a selective biosensor, and differentiated between Gram-positive and negative bacteria based on fluorescence. Practicability was tested by detecting lysozyme in human urine and serum samples with good recovery rates, with the fluorescence quenching attributed to competitive interactions between lysozyme and the nanoclusters. In differentiating bacteria according to Gram stain character, the nanocluster showed no fluorescence with Gram-negative bacteria; however, it emitted strong fluorescence when interacting with Gram-positive bacteria. Further evaluations indicated low toxicity, showing promise as bioprobes, however, leaving room for improving the observed low yield of the nanoclusters and enhancing selectivity through signal amplification strategies.

*Cronobacter sakazakii*, a foodborne pathogen of major concern in powdered infant formula, poses serious risks to infants, yet existing detection methods are slow and insufficiently sensitive. Thus, Kang *et al.*^75^ developed a fluorescent biosensor that couples the aggregation-induced emission properties of gold nanoclusters with dual nucleic acid amplification *via* exponential amplification reaction (EXPAR) and hybridisation chain reaction (HCR). Using pathogen-specific hairpin probes, the system triggered G-quadruplex formation and measurable fluorescence changes, enabling ultrasensitive detection down to ∼1.10 CFU mL^−1^. The study confirmed the specificity of the biosensor against other foodborne bacteria, with recovery rates near 100%. The findings suggest that this system could provide a rapid, selective, and cost-effective diagnostic platform with promising applications for food safety monitoring and potential adaptability to other pathogens.

For therapeutic purposes, Wang *et al.*^76^ demonstrated improved antibacterial and wound healing properties using a biocompatible hydrogel. These hydrogels were composed of silver nanoclusters (AgNCs) loaded with lysozyme, curcumin, and crosslinked with calcium ions. The resulting LC-AgNCs exhibited distinct fluorescence properties, were stable, and uniform. They also had a good gelation rate and mechanical strength, necessary for wound application. LC-AgNCs showed a dose-dependent antibacterial property against *E. coli* and *S. aureus* while reducing bacterial viability over time. The antibacterial effect was attributed to bacterial cell membrane disruption/damage due to depolarization and intracellular leakages. It also showed promises of supporting tissue regeneration during wound healing. This was elucidated in an *in vivo* mouse model, where the nano cluster significantly accelerated wound healing, as indicated by histological analysis, which showed reduced inflammation and enhanced tissue regeneration.

More recently, Dai *et al.* developed Au_12−*x*_Ag_*x*_ alloy nanoclusters (*x* = 4, 5, 6), for light-responsive treatment of infected wounds. The study showed that changing the Au/Ag composition altered the electronic and redox properties of the nanoclusters, with increasing silver incorporation enhancing photocatalytic generation of hydroxyl radicals under ultraviolet irradiation. Among the investigated formulations, Au_6_Ag_6_ demonstrated the strongest photocatalytic activity and achieved 99.9% antibacterial efficacy against both Gram-positive and Gram-negative bacteria. The nanoclusters were incorporated into wound dressings and activated by UV light, and their antimicrobial property was tested in an *S. aureus*-infected mouse wound model. The Au_6_Ag_6_ nanoclusters achieved 77.4% bacterial clearance by day 3 as well as complete elimination of recoverable bacteria by day 11. By day 11, the residual wound area was only 0.9%, compared with 2.2% for Au_7_Ag_5_ + UV and 6.6% for Au_8_Ag_4_ + UV. The Au_6_Ag_6_ treatment also produced 83.6% collagen deposition and reduced IL-1β-positive cells from 32.7% in the control group to 4.8%, indicating improved tissue regeneration and reduced inflammation.^36^

Murugan *et al.*^77^ studied how different thiol ligands influence the properties of thiol-protected iron nanoclusters (FeNCs). The study focused on their optical behaviour, antibacterial potential, and biocompatibility. The authors synthesized FeNCs using glutathione (GSH), 3-mercaptopropionic acid (MPA), and 3-mercaptopropane sulfonate (MPS), and characterized them using UV-Vis spectroscopy, XRD, HRTEM, AFM, and PAGE separation. The study observed distinct absorption peaks and crystalline features, which confirmed successful synthesis and the presence of ligands. Photoluminescence assays showed FeGSH and FeMPA NCs with higher quantum yields (5.6 and 5.8%) compared to FeMPS (2.1%), highlighting the importance of ligand structure. Cell viability tests on MCF-7 cells indicated good biocompatibility, while antibacterial assays demonstrated activity against multiple bacterial strains, with FeGSH NCs specifically effective against Gram-negative bacteria. The findings reveal that the choice of ligand significantly affects the functional properties of FeNCs, thus providing insights for their biomedical application.

Overall, the mechanisms employed by the various nanoclusters range from the generation of ROS, membrane disruption, phototherapy, to gene sensing and detection, as observed respectively with AgNCs, CuNCs, DNA-CuNCs, and AuNCs (Table 1). They are, however, apparently limited by non-specific oxidative stress, possible impact on bacterial host cells, poor stability in serum, and light penetration limits, respectively. Also, most current studies lack *in vivo* infection models, with few studies using clinical isolates, inadequate reports on safety, and no head-to-head comparisons with antibiotics. Thus, there is an apparent need for research driven in these directions, including the consideration of regulatory engagements and GMP scale-up.

**Table 1:** Recent studies on the use of nanoclusters in the diagnosis and treatment of bacterial infections

Composition	Method of synthesis	Target bacteria	Mechanism of action	Key finding	Ref.
Glutathione-protected silver nanoclusters (GSH-Ag-R NCs)	Cyclic NaBH_4_ reduction–decomposition of AgNO_3_/GSH complexes	Gram-negative *P. aeruginosa*, *E. coli*; Gram-positive *B. subtilis*, *S. aureus*	GSH-Ag-R NCs disrupt membranes and release Ag^+^; GSH-Ag^0^ R NCs kill *via* slow Ag^+^ release	GSH-Ag R NCs inhibit bacteria at lower MICs (80–100 µM) than GSH-Ag^0^ R NCs, comparable to ampicillin	78
Lysozyme-stabilized gold nanoclusters conjugated with rose bengal (Lys-Au NCs/RB)	One-pot reduction of HAuCl_4_ with lysozyme at alkaline conditions	*S. mutans*, *E. coli*, *A. naeslundii*, *P. gingivalis*	ROS production and intrinsic lysozyme antibacterial activity in Lys-Au NCs/RB cause killing of planktonic bacteria and damage to *S. mutans* biofilms	Photoexcited Lys Au NCs/RB enhance antibacterial effects with low toxicity, ideal for photodynamic therapy	79
Electrochemically synthesized silver ultra-nanoclusters ARGIRIUM-SUNCs®	Electrochemical generation of silver ultra-nanoclusters in ultrapure water	*P. syringae*, *X. vesicatoria*, *X. fastidiosa*, *C. michiganensis*	Ag^2+^/Ag^3+^ species inhibit growth, biofilm formation, and eradicate established biofilms through strong cell interactions	ARGIRIUM-SUNCs show strong antibacterial, antibiofilm activity and 80% protection against bacterial speck without phytotoxicity	80
BSA-stabilized gold nanoclusters (BSA-AuNCs)	BSA-templated biomineralization	*K. pneumoniae*, *A. baumannii*, *E. coli*, *P. aeruginosa*, *P. mirabilis*	BSA AuNCs detect carbapenemase-producing cells with fluorescence change, enabling detection down to 10 CFU mL^−1^	BSA AuNC assay accurately detects CROs with 100% PPV/NPV, outperforming Carba NP test, offering rapid diagnostics	81
Antimicrobial peptide-functionalized gold nanoclusters	One-step reduction using cysteine-containing AMPs and Au^3+^ (from HAuCl_4_)	*S. aureus*, *S. epidermidis*, *S. pneumoniae*; *E. coli*, *A. baumannii*, *P. aeruginosa*; *E. coli*	Electrostatic and hydrophobic interactions between each AMP-AuNC and bacterial surfaces modulate fluorescence	AMP AuNC sensor array achieves 100% identification accuracy for reference bacteria and 86.7% for clinical *E. coli* UTI isolates using machine learning	73
Riboflavin-protected ultrasmall silver nanoclusters	Reduction of AgNO_3_ by riboflavin	*S. aureus*, *E. coli*, and the fungus *C. albicans*	RF@AgNCs disrupt bacterial cell membrane integrity and induce high levels of intracellular reactive oxygen species, leading to the death of bacteria and fungi	RF@AgNCs show stronger antibacterial/antifungal activity than AgNPs, with negligible hemolysis and low cytotoxicity	82
Bacitracin-directed silver, gold, and copper nanoclusters	One-pot reduction process using bacitracin AgNO_3_, HAuCl_4_, or CuSO_4_	*S. aureus* (MRSA, ATCC 6538)	Combining metal nanoclusters with bacitracin in a single construct enhances ROS generation, leading to potent killing of *S. aureus*	AgNCs@bacitracin inhibits MRSA growth and accelerates wound healing in infected mice	83
Conjugated-polymer nanoparticles coated with AMP and gold nanoclusters (CNP@AMP-AuNCs)	Nanoprecipitation, EDC/NHS coupling for covalent attachment of the AMP, and reduction/stabilization of gold nanoparticles (AuNCs)	*E. coli*	AMP shell disrupts bacterial membranes, AuNCs enable imaging, and CNP core facilitates photothermal ablation of cancer cells	CNP@AMP-AuNCs kill *E. coli* and cancer cells, demonstrating a multifunctional nanoplatform for infection and cancer treatment	84
Silver nanocluster/silica composite coating on Kevlar® fabric	Radio-frequency co-sputtering of SiO_2_ (RF 200 W) and Ag (DC 1 W) onto Kevlar®	*S. aureus*, *E. coli*, and the fungus *C. albicans*	Ag nanoclusters embedded in silica gradually release Ag^+^ in aqueous and sweat media, producing local inhibition zones and reducing bacterial adhesion on coated fibers	Ag-nanocluster/silica-Kevlar® exhibits broad-spectrum antibacterial activity, ideal for antimicrobial textiles in space habitats	85
Glutathione-protected silver nanoclusters	GSH-capped Ag nanoclusters *via* reduction of AgNO_3_/GSH complexes; Ag NC-MSNs *via* Stöber-type condensation of TEOS	*S. aureus* (ATCC 6538), *P. aeruginosa* (ATCC 27853)	Strong interaction with bacterial cell-wall biomolecules, membrane damage, ROS generation, and intracellular Ag^+^ uptake, leading to enzyme dysfunction and cell death	Ag NC-MSNs show higher bactericidal potency than Ag NCs and Ag-NP-MSNs with minimal toxicity to HeLa cells	86
Bovine serum albumin-stabilized gold nanoclusters (BSA@Au)	One-pot aqueous reduction of HAuCl_4_ using BSA	Methicillin-resistant *S. aureus* (MRSA)	BSA@Au binds strongly to MRSA and generates singlet oxygen from H_2_O_2_, causing membrane damage and complete bacterial killing in hypoxic infection microenvironments while enabling NIR-II fluorescence imaging of infected sites	BSA@Au shows intense fluorescence for *in vivo* imaging, negligible systemic toxicity, eradicates MRSA *in vitro*, and markedly accelerates healing of MRSA-infected wounds in mice	67
Thiol-protected copper nanoclusters (CuNCs)	Wet-chemical reduction of a Cu(ii) salt in the presence of aromatic/aliphatic thiol ligands to form CuNCs	*E. coli* and *S. aureus*	CuNCs release Cu^+^/Cu^2+^ and interact with bacterial membranes, leading to membrane damage, leakage of intracellular components, and oxidative stress, leading to death	Thiol-protected CuNCs exhibit notable antibacterial activity against both *E. coli* and *S. aureus*, with low MIC values and clear inhibition zones	72
Papain-encapsulated platinum nanoclusters (papain-Pt NCs)	One-pot aqueous synthesis where papain acts as both template and reducing/stabilizing agent for Pt^2+^	Lysozyme in biofluids, Gram-positive *vs.* Gram-negative bacteria for indirect differentiation	Lysozyme binding to papain-Pt NCs enables fluorescence-based distinction of Gram-positive and Gram-negative infections	Papain-Pt NCs selectively detect lysozyme, distinguishing Gram-positive/negative bacteria *via* fluorescence changes for bacterial typing	74
DNA-templated gold nanoclusters	Amplified fluorescence biosensing	*C. sakazakii*	*C. sakazakii* DNA triggers EXPAR-HCR, forming G-quadruplexes that induce AuNC fluorescence for selective detection	Biosensor detects *C. sakazakii* at 1.10 CFU mL^−1^ with reprogrammable specificity	75
Hydrogel containing lysozyme, curcumin, and silver nanoclusters (LC-AgNCs)	Composite hydrogel fabrication	*E. coli* and *S. aureus*	Lysozyme disrupts peptidoglycan, curcumin offers antimicrobial/anti-inflammatory effects, and Ag nanoclusters release Ag^+^ ions in hydrogel	LC-AgNC hydrogel showed clear, dose-dependent antibacterial efficacy against both *E. coli* and *S. aureus* and significantly reduced bacterial viability over time	76
Thiolate-protected iron nanoclusters	Reduction of an iron salt in the presence of each thiol ligand	*E. coli* and *S. aureus*	Membrane disruption leading to bacterial death	Thiol ligand choice modulates optical and biological behavior of the nanoclusters: all three formulations are biocompatible, fluorescent, and antibacterial	77
DNA-templated silver nanoclusters (AgNCs)	Isothermal amplification coupled with Cas12a/crRNA recognition and collateral cleavage	*S. typhimurium*	Cas12a cleaves AgNC-labeled ssDNA, altering fluorescence for *S. typhimurium* detection	SCENT-Cas enables ultrasensitive, selective *S. typhimurium* detection with 1 CFU mL^−1^ limit	69
Hairpin DNA-stabilized copper nanoclusters (CuNCs)	One-step reduction	*C. trachomatis*	Hairpin DNA opens upon hybridization, altering CuNC fluorescence for detection	Hairpin-CuNC probe enables label-free, sequence-specific bacterial DNA detection *via* fluorescence change	70

### 3.2 Nanoclusters for parasitic diseases

Parasites such as *Plasmodium* spp. (malaria), *Leishmania* spp. (leishmaniasis), *Trypanosoma* spp. (trypanosomiasis), *Toxoplasma gondii* (toxoplasmosis), *Schistosoma* spp. (schistosomiasis), and helminths significantly contribute to the global disease burden, particularly in low- and middle-income countries. While there are current management options, they, however, have varying degrees of limitations. Although not many experimental procedures have been conducted, nanoclusters, with tunable photoluminescence, ultra-small size, and quantum effect characteristics, show promise for effective control, diagnosis, management, and treatment of parasitic diseases, with recent advances beginning to illuminate their translational potential.

Wang *et al.*^87^ aimed at improving the diagnosis of malaria by specifically detecting the key malaria biomarker, *Plasmodium falciparum* lactate dehydrogenase (PfLDH), using DNA-scaffold silver nanoclusters (AgNCs-dsDNA) in combination with a specific DNA aptamer for PfLDH, while the DNA stabilises and enhances the luminescence properties of the silver nanoclusters. The study showed that AgNCs-dsDNA significantly enhanced luminescence compared to single-stranded AgNCs, producing a quantum yield of 5.36%. Additionally, they reported a significant increase in fluorescence intensity following the addition of PfLDH, with a 12-fold enhancement at a concentration of 1.50 µM and a limit of detection of 0.2 nM, which is clinically relevant for detecting PfLDH in plasma (Fig. 4). It was specific to PfLDH, displaying weaker responses and fluorescence enhancement when exposed to *Plasmodium vivax* lactate and human lactate dehydrogenase. With a two-step fluorescence response recorded with PfLDH, they proposed that this is due to interactions at different binding sites on PfLDH, with the G-rich strand in the complementary oligonucleotide playing an important role in enhancing fluorescence. Preclinically, using fetal calf serum, they demonstrated the feasibility of the diagnostic nanocluster by detecting PfLDH, achieving a limit of detection of 1.0 nM, thus supporting clinical application while leaving room for optimisation, especially in improving selectivity against other lactate dehydrogenases.

**Fig. 4:**
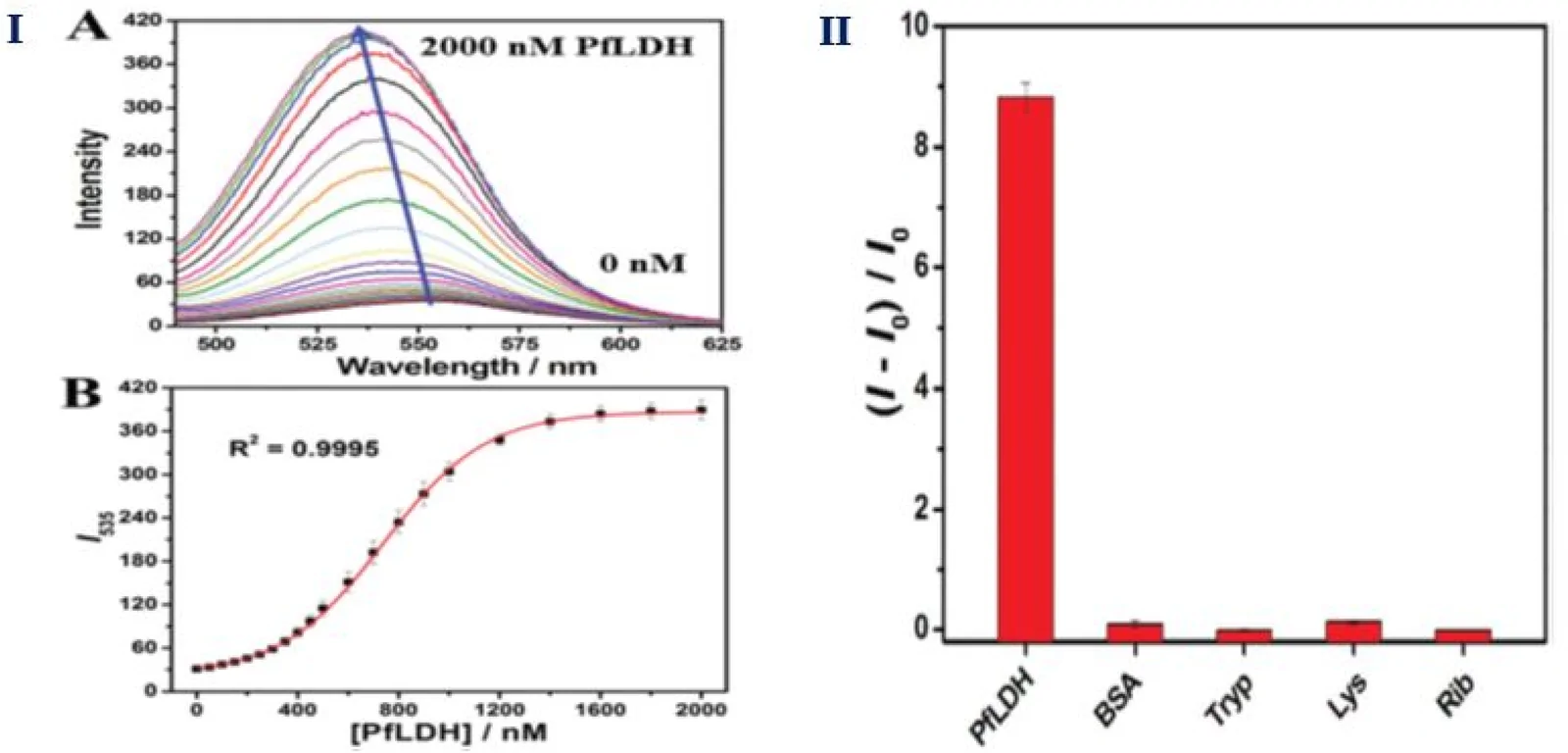
Sensitivity and specificity of DNA-scaffolded silver nanoclusters for malaria diagnosis: (I) fluorescence enhancement of AgNCs-dsDNA upon PfLDH addition, showing up to a 12-fold increase in intensity and a clinically relevant detection limit of 0.2 nM; (II) comparative fluorescence responses of AgNCs-dsDNA to PfLDH, PvLDH, and human LDH, demonstrating strong selectivity toward PfLDH. Reproduced from ref. 87 with permission from The Royal Society of Chemistry.

Expanding on this approach, Zhang *et al.*^88^ sought to improve the sensitivity and selectivity of *Plasmodium vivax* lactose dehydrogenase (PvLDH) and employed adenosine monophosphate-protected gold–silver bimetallic nanoclusters (Au–AgNCs@AMP) in designing a fluorescent probe. Both Wang *et al.* and Zhang *et al.* developed advanced fluorescence sensors for detecting malaria biomarkers. While Wang *et al.* focuses on high-specificity, aptamer-based detection, Zhang *et al.* expanded their study to a bimetallic Au–AgNC “aptamer-free” system for *P. vivax* (PvLDH), providing a dual preventive and detection purpose. Upon titrating different concentrations of PvLDH into Au–AgNCs@AM, they reported an increase in fluorescence intensity, with a maximum enhancement at 1.0 µM PvLDH and the emission peak moving from 550 to 500 nm due to changes in the microenvironment of the nanoclusters. They also demonstrated a low limit of detection (0.10 nM) and a linear relationship between the concentration of PvLDH and fluorescence intensity, thus indicating the potential for quantitative detection over a range of concentrations (10–100 nM). In terms of selectivity, the nanocluster produced a significantly stronger fluorescent response to PvLDH whilst discriminating against other introduced proteins, particularly after the introduction of Al^3+^ into the system. *In vivo*, the authors also effectively detected PvLDH in fetal calf serum with recovery rates between 99.3 and 102%. The mechanism behind the enhanced fluorescence was attributed to electrostatic interactions, with confirmed changes in the nanocluster's surface electron energy resulting from these interactions, and thiol groups in PvLDH playing significant roles in the process.

Beyond diagnostics, nanoclusters have demonstrated therapeutic potential, Wang *et al.*^89^ targeted *Trypanosoma brucei*, a causative agent of African trypanosomiasis, using Ag_2_S-NC@MPA and AuNC@GSH, two nanoclusters, composed of silver sulfide (Ag_2_S) with 2-mercaptopropionic acid (MPA) (surface ligand) and gold (Au) with glutathione (GSH) (surface ligand), respectively, for their endocytotic activities, using cytometry, microscopy, and proteomic analysis. The authors reported the concentration- and time-dependent effects of the metal nanoclusters and showed that clathrin plays essential roles in the uptake process. Cell viability studies showed that the nanoclusters induced apoptosis in a concentration-dependent manner, with Ag_2_S-NC@MPA having the best apoptotic effect. Oxidative stress and mitochondrial dysfunction were reported as key mechanisms for the observed cytotoxicity following the confirmation of the significant alteration of the levels of calcium ions, reactive oxygen species, and mitochondrial membrane potential. From proteomic and metabolomic analysis, Ag_2_S-NC@MPA was reported to affect the mitochondrial function and trypanothione metabolism, while AuNC@GSH had a significant impact on glycolysis and energy metabolism.

Drug solubility and resistance remain critical barriers for helminthic infections, with albendazole, a broad-spectrum anthelmintic, often limited by its variable bioavailability and water solubility, subsequently contributing to its resistance by gastrointestinal nematodes and therapeutic uses, with economic impacts on livestock. To address this, Pacheco *et al.*^90^ evaluated the solubility and anthelminthic efficacies of the drug formulated with β- and hydroxypropyl-β cyclodextrins, with and without polyvinylpyrrolidone (PVP). Following complexation with the nanoclusters, the hydroxypropyl-β cyclodextrins exhibited the highest solubility, with PVP further enhancing the solubility and dissolution rates, thus suggesting the combination to have the potential to improve drug delivery. However, while the solubility was generally enhanced, the formulations did not present any significant enhancement in efficacy compared to the pure drug. This was confirmed by treating lambs naturally infected with gastrointestinal nematodes and recording/comparing faecal egg count reduction before and after treatment, with indicative *Haemonchus* spp. maintaining its level across the treatment groups, thus revealing complexities in the formulation, including rapid biotransformation of the drug into its inactive metabolite.

A similar strategy was employed by Rodrigue *et al.*^91^ using organic nanoclusters of cyclodextrins to investigate the enhancement of the solubility of albendazoles and fenbendazoles, two derivatives of benzimidazoles. Specifically, they complexed the drugs with β-cyclodextrin and hydroxypropyl-β-cyclodextrin, and in combinations with polyvinyl pyrrolidone (PVP) as a co-complexing agent and showed the increase in the solubility of albendazole from 0.4188 µg mL^−1^ to 93.47 µg mL^−1^ and 443.06 µg mL^−1^ with β-cyclodextrin and hydroxypropyl-β-cyclodextrin, respectively and the solubility of fenbendazole increased from 0.1054 µg mL^−1^ to 45.56 and 159.36 µg mL^−1^, respectively. Also, they demonstrated that PVP enhanced the amorphisation of the drug/nanocluster system, which is beneficial to solubility. Synergistically, cyclodextrin and PVP further enhanced the dissolution rates compared to the pure drugs, with hydroxypropyl-β-cyclodextrin being the most effective for optimising the solubility of the drugs. Pacheco and Rodrigues teams developed supramolecular organic nanoclusters aimed at overcoming the poor aqueous solubility of benzimidazole anthelmintics, specifically albendazole and fenbendazole using cyclodextrin complexation. The study by Rodrigues and his team is a fundamental *in vitro* characterization that explores the physical chemistry and structural physics of drug–carrier interfaces. In contrast, Pacheco *et al.* conducted a translational *in vivo* veterinary trial to measure actual therapeutic efficacy in animals. Despite the increase in *in vitro* drug solubility, the *in vivo* clinical efficacy did not differ significantly from that of pure albendazole. Further studies are needed to ascertain the intestinal permeability and stability of the complexed drugs.

The application of nanoclusters (Table 2) in parasitology is emerging but currently underserved. However, if properly designed, it can fill critical gaps. Major gaps identified include the lack of cross-species and disease nanocluster platforms, the absence of *in vivo* kinetic studies, poor target engagement due to parasite state-specific variations, and limited field validation, which leads to unrealistic use cases. While further developments are necessary, there is also a need to thoroughly elucidate the pharmacokinetic and pharmacodynamic validation, prioritising cross-parasite platform versatility, scalability, and clinical integration, alongside mechanistic studies that bridge bench-to-bedside translation. Additionally, ensuring stability in biological fluids such as lysosomes, the gut, and serum, as well as achieving selective detection or response in parasite-protected niches such as vacuoles, hepatocytes, and the central nervous system (CNS), is essential. With these in place, we propose that nanoclusters can be modularised into plug-and-play theranostic kits that detect and neutralise parasitic pathogens in real-time, particularly in resource-limited settings.

**Table 2:** Recent studies on the use of nanoclusters in the diagnosis and treatment of parasitic infections

Nanoclusters	Code	Effects	References
DNA-scaffold silver nanoclusters in combination with a specific DNA aptamer	AgNCs-dsDNA	Significantly enhanced luminescence compared to single-stranded AgNCs. Significant increase in fluorescence intensity with specific detection of PfLDH	87
Adenosine monophosphate-protected gold–silver bimetallic nanoclusters	Au–AgNCs@AMP	Increase in fluorescence intensity, with a maximum enhancement at 1.0 µM PvLDH. Low limit of detection and a linear relationship between the concentration of PvLDH and fluorescence intensity. Significantly stronger fluorescent response to PvLDH. *In vivo*, detection of PvLDH in fetal calf serum	88
Silver sulfide (Ag_2_S) with 2-mercaptopropionic acid (MPA) (surface ligand) and gold (Au) with glutathione (GSH) (surface ligand)	Ag_2_S-NC@MPA and AuNC@GSH	Reported time-dependent concentration of the metal nanoclusters. Induced apoptosis in a concentration-dependent manner. Ag_2_S-NC@MPA affected the mitochondrial function and trypanothione metabolism, while AuNC@GSH had a significant impact on glycolysis and energy metabolism	89
Albendazole formulated with β- and hydroxypropyl-β cyclodextrins, with and without polyvinylpyrrolidone	—	Exhibited high solubility, though no significant enhancement in efficacy compared to the pure drug	90
Albendazoles and fenbendazoles complexed with β-cyclodextrin and hydroxypropyl-β-cyclodextrin, and in combinations with polyvinyl pyrrolidone (PVP)	—	Increase in the solubility of albendazole and fenbendazole. Demonstrated that PVP enhanced the amorphisation of the drug/nanocluster system. Synergistically, cyclodextrin and PVP further enhanced the dissolution rates compared to the pure drugs	91
NanoCluster Beacons (NCBs) in Rolling Circle Enhanced Enzyme Activity Detection (REEAD) assays	NCBs/sNCB	Cost-effective, photostable NCBs with enhanced fluorescence and sensitivity, outperforming organic dyes; effective in detecting human topoisomerase I and validated in *Plasmodium*, with potential for malaria diagnostics and theranostics	92

### 3.3 Nanoclusters for viral diseases

Over the years, viral infections have posed significant threats to human health, as seen with the recent COVID-19 pandemic.^93–95^ These infections can cause high morbidity and mortality. They also put a lot of strain on the healthcare systems and disrupt economies, emphasizing the need for more effective diagnostic and therapeutic tools for tackling these diseases.^96^ As such, nanoclusters (NCs) have gained attention as promising candidates for the management of various viral diseases. Their ultrasmall size, typically below 10 nm, provides discrete electronic states and strong fluorescence signals, properties that are particularly valuable to ensure highly sensitive viral detection and targeted drug delivery.^97^ Moreover, their tunable surface chemistry allows easy conjugation with biomolecules, enabling selective recognition of viral proteins or nucleic acids.^98^ Together with their straightforward synthesis, cost-effectiveness, and favorable biocompatibility, these attributes make metal nanoclusters versatile platforms for the diagnosis and treatment of viral infections.

Kurdekar *et al.* developed ultrasensitive fluorescent sensors for early diagnosis of HIV infection and other viral infections.^99^ These 2 nm AuNCs exhibited high fluorescence, as evidenced by UV-Vis and photoluminescence studies. As shown in Fig. 5, antibodies were coated on the microtiter wells that capture the HIV-1 p24. The addition of biotinylated anti-p24 antibodies resulted in their binding to the captured p24 antigen. The complex then attaches to the streptavidin-conjugated gold nanoclusters, which then interact with the biotinylated complex. After multiple wash steps, the signal released from the wells was recorded. The nanoclusters were highly fluorescent and emitted at 615 nm (excitation at 350 nm).^99^ Computational analyses revealed an enhanced binding affinity between the AuNCs and specific active sites. Notably, when tested against HIV-1-p24 antigen in clinical samples, these streptavidin-conjugated AuNCs demonstrated significantly higher sensitivity and specificity, surpassing the enzyme-linked immunosorbent assay by threefold.^99^

**Fig. 5:**
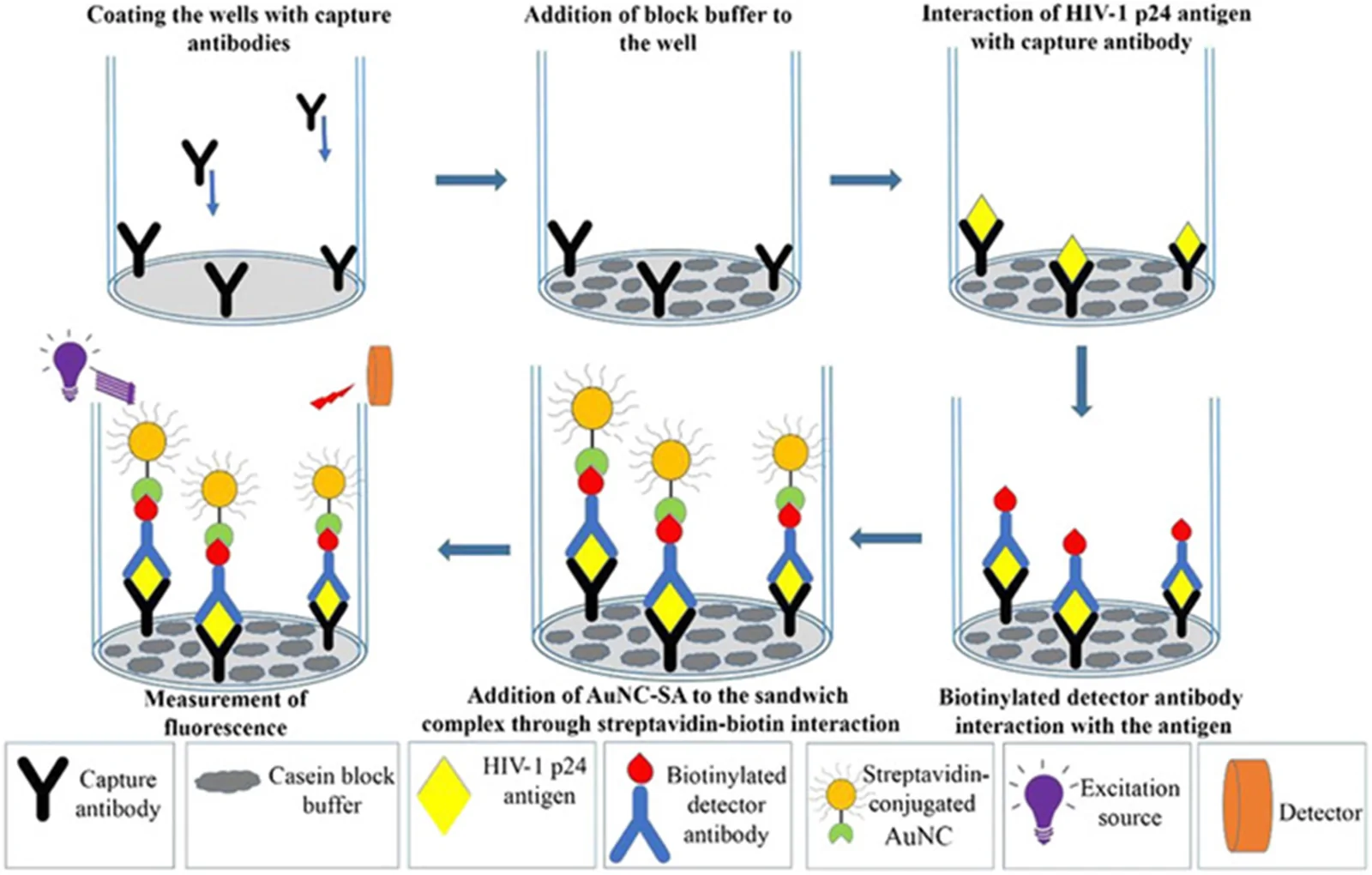
Schematic representation of streptavidin-conjugated gold nanoclusters for the detection of HIV-1 p24 antigen. Reproduced from ref. 100 with permission from The Royal Society of Chemistry.

In another study, scientists reported a novel immunoassay for detecting SARS-CoV-2 antibodies and viruses using bovine serum albumin-protected AuNCs etched with cysteamine.^101^ For virus detection, they targeted the SARS-COV-2 virus nucleocapsid protein (NP) by immobilizing antibodies on magnetic beads to capture NP. Detection was then achieved using rabbit anti-SARS-COV-2 nucleocapsid antibodies and alkaline phosphatase (AP)-conjugated antibodies. A similar approach was employed to assess SARS-COV-2 antibody levels by capturing spikes in SARS-COV-2 receptor-binding domain (RBD)-specific antibodies using RBD-modified magnetic beads. In this case, detection was performed using AP-conjugated anti-human IgG antibody. The sensing mechanism for both immunoassays rely on cysteamine etching-induced fluorescence quenching of bovine serum-protected AuNCs. Cysteamine was generated in proportion to the amount of either SARS-CoV-2 virus or anti-SARS-CoV-2 receptor-binding domain-specific immunoglobulin antibodies (Fig. 6). Gold nanoclusters (AuNCs) have demonstrated high sensitivity in immunoassays, offering quick results and a nanogram-level limit of detection (LOD). These techniques can be applied clinically to detect coronavirus during active infection or to identify antibodies post infection.^101^ The versatility of AuNCs in viral infection diagnosis is promising owing to their adaptability to various analytes. Although still in the research phase, AuNCs show potential for development into a universal labeling method for precise viral antigen detection at the nanogram or picogram levels.

**Fig. 6:**
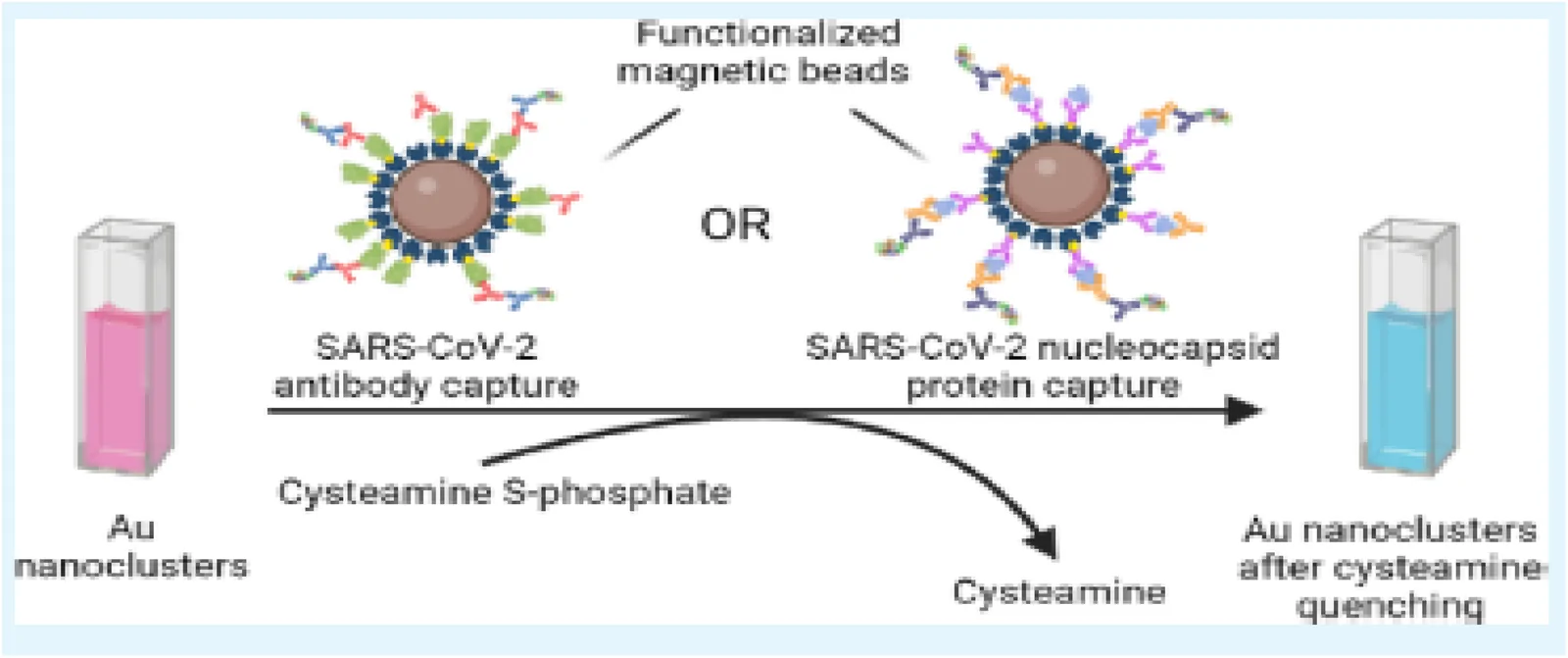
Sensing mechanism based on cysteamine etching-induced fluorescence of bovine serum albumin-protected gold nanoclusters. Reproduced from ref. 101 with permission from American Chemical Society.

Metal nanoclusters have also been explored for the diagnosis of hepatitis B virus (HBV). Traditional clinical practice often relies on PCR for HBV detection. This method however, is costly, time-consuming, and requires specialized skills. Nanoclusters offer a potential solution because NC-based diagnostic techniques are simple and cost-effective. Researchers have developed innovative and sensitive fluorescent biosensors for the detection of HBV. This sensing system incorporates CRISPR-Cas12a enzymes and metal nanoclusters as luminescent nanoprobes, involving gold nanoclusters (AuNCs), silver nanoclusters (AgNCs), and copper nanoclusters (CuNCs) without labeling.^102^ The DNA probes were designed to be a template for the formation of the metal nanoclusters. In the absence of the HBV target, NCs were formed and presented with strong fluorescence signals, while in the presence of the HBV target, formation of NCs was inhibited. This mechanism of action was based on the trans-cleavage ability of the CRISPR-Cas12a enzyme to break down the DNA probes which resulted in inhibition of the formation of nanoclusters seen as weak fluorescence signals in the presence of the HBV moiety. However, when the 3 metal NCs were compared, the CuNCs showed greater and faster sensitivity in HBV detection. The use of CuNCs as nanoprobe for Cas-12 assisted detection of HBV-DNA is a promising approach in the diagnosis of hepatitis B diseases.^102^ At clinical levels this facile means of HBV detection using NCs could be deployed to our laboratories, hospitals and health centers to save cost and time.

Aside from the diagnostic potentials of metal NCs for viral organisms, drug delivery or targeting uses has also been reported. A researcher produced a composite coating composed of silver NCs embedded in silica matrix and deposited it onto glass fibres and polymeric air filters by the cosputtering technique. An in-depth understanding of the possible virucidal activity against the human coronavirus OC43 strain A as well as the possible mechanism of action was investigated.^103^ The release of silver ions from the coating preparation was dependent on the size of the NCs, the concentration and the nature of the matrix where the silver NCs were embedded.^104^ In this study, silver ions were embedded in the silica matrix, which is porous in nature. This allowed the silver ions to be released from the embedded NCs. When the virucidal effect was investigated, there was a significant reduction (0.8 logarithm of inactivity) in the number of infectious coronavirus particles compared the uncoated glass fibers. In addition, a possible mechanism of action was predicted based on the interaction of the silver NCs and the materials of the air filter and glass fibers. For the polymeric air filters, the coating directly altered and inactivated the viral particles, this interaction was independent of the release of silver ions. On the one hand, antiviral effect of the silver NCs deposited on the glass was mediated by silver ion release from the coating.^103^ Similarly, another research report in the heat of the COVID-19 infection showed the use of silver nanocluster coating on facial FFP3 mask to protect against the coronavirus infection especially for health workers at front line.^105^ Their preliminary investigation showed that thin coating of the silver NCs on the non-woven fabric of facial mask was able to elicit profound virucidal effect against clinical isolate of the virus. However, the extent of virucidal effect was based on concentration of the silver ions used. Higher concentration of silver NCs proved completely virucidal while lower concentration showed some degree of virus clearance compared to the uncoated facial mask.^105^ Clinically, silver NCs can be deployed in the purification of air, especially for indoor environments. A decrease in air quality is usually propagated by airborne pollutants (bacteria, virus or fungi) which could predispose to a plethora of health problems including infectious diseases. Therefore NCs formulated as coating agents deposited on personal protective equipment such as facial masks or air filters found in public places or on surfaces can play a useful role in reducing the incidence of viral diseases, ensuring clean air, decreasing the extent of the infectious cycle, and promoting a disease-free environment. NCs can be used in actively targeting viruses or virus-like particles. Marjomäki *et al.* described the functionalization of AuNCs with maleimide linkers to target cysteine residues on the capsid proteins of two enteroviruses, echovirus 1 (EV 1) and coxsackievirus B3 (CV B3) through covalent bonds. Their findings revealed that the addition of NCs enhanced the structural appearance of viruses, when examined by transmission electron microscopy (TEM). Furthermore, the viruses maintained their infectivity regardless of the degree of AuNCs conjugation. The strong covalent attachment of NCs to viruses offers the potential for *in vivo* viral studies. For instance, this method can be employed to investigate or visualize the infectious pathways of enteroviruses from the intestine to target tissues. In clinical applications, conjugation of NCs to attenuated viruses used as vaccines could provide crucial insights into their cellular and tissue entry mechanisms. In summary, NCs have tremendous potential as a facile diagnostic tool for virus detection and a great virucidal agent to reduce the burden of viral diseases (Table 3).^106^

**Table 3:** Recent studies on the use of nanoclusters in the diagnosis and treatment of viral infections

Type of nanocluster	Experimental model/organism	Findings	Potential use	Ref.
DNA/silver nanoclusters probes	Citrus Tristeza virus	5-Fold enhancement in target sensitivity	Selective DNA probe for the detection of Tristeza mild strains	107
		Effectively differentiated between mild and severe RNAs		
		Reduced fluorescence emission ratio in the presence of target RNAs		
		*I*_0_/*I* intensity ranged from 1.5 × 10^−8^ M to 1.8 × 10^−6^ M		
		The limit of detection was 4.3 × 10^−9^ M		
DNA-templated copper nanoclusters	Hepatitis B virus	Sensitive	Cost-effective colourimetric detection of hepatitis B virus DNA	108
		The limit of detection was 12 × 10^9^ molecules		
Glutathione (GSH)-capped silver nanocluster	Porcine epidemic diarrhoea virus (PEDV)	Significant suppression of PEDV infection with a 3.0 log decrease in virus titer 12 h post-infection	Potential therapeutic agent for coronavirus	109
		Inhibition of the synthesis of viral negative-strand RNA and viral budding		
		Activation of IFN-stimulating genes (ISGs) generation		
		Upregulation of pro-inflammatory cytokine expression		
Glutathione-stabilised fluorescent gold nanoclusters	Pseudorabies virus (PRV) and porcine reproductive and respiratory syndrome virus (PRRSV)	Selective inhibition of proliferation and protein expression of PRRSV (an RNA virus)	Specific antiviral agent against RNA virus infections	110
		Direct inactivation of PRRSV and blockage of viral adsorption		
		No effect on PRV (a DNA virus) proliferation and protein expression		
		Viral genome replication was unaffected		
Streptavidin-conjugated gold nanoclusters	HIV-1 p24 antigen	Formed through the chemical reduction of chloroauric acid	Ultrasensitive fluorescent sensors for early diagnosis of HIV infection and other viral infections	99
		Nanoclusters were highly fluorescent and emitted at 615 nm (excitation at 350 nm)		
		Enhanced binding to the protein *in silico*		
		Limit of detection of 5–1000 pg mL^−1^		
		Threefold higher sensitivity and specificity compared to the enzyme-linked immunosorbent assay		
		100% specificity obtained from assay with negative for HIV-1 p24 antigen negative plasma samples		
Iron–phenanthroline nanocluster loaded with mesoporous curcumin	HIV infected human microglia	Downregulation of HIV-p24 by 41%	Adjuvant HIV therapy with traditional anti-retroviral regimens	111
		Reduced *in vitro* expression of pro-inflammatory mediators such as TNF-α, IL-8 and NO		
		Upregulation of catalase CAT and downregulation of hemeoxygenase-1 HMOX-1 gene expression		
Virus capsid protein (CP25) copper nanoclusters	*E. coli* expressed – CP25	CP25 protein was used as a target and a tracer	Tracer for the competition fluorescence anisotropy immunoassay for the detection of Citrus Tristeza virus	112
	Citrus Tristeza virus	Quantum dot-like behaviour observed		
		Light emission at 535–715 nm		
		Amplification of the anisotropy signal due to interaction with anti-Citrus Tristeza virus antibody		
		An increase in CP25 protein levels led to a decrease in FA values from 400 pg mL^−1^ to 25 ng mL^−1^		
		The limit of detection was 220 pg mL^−1^		
		Reduced protein affinity but did not hinder antibody binding		
Copper nanoclusters with CRISPR-Cas12a enzymes	Hepatitis B virus	Superior sensitivity to silver and gold nanoclusters	Rapid biosensor for hepatitis B virus DNA detection	113
		Rapid response (within 25 min) to hepatitis B virus DNA detection		
Peptide–gold nanocluster-assembled binder (PEGNAB)	Influenza A virus	High affinity for influenza A virus spike protein, hemagglutinin	Ultra-high sensitivity PEGNAB-based antigen assay kit and paper strip for the detection of influenza A virus	114
		Approximately 500–800 PEGNAB particles were bound per virion		
		The linear detection range for influenza A virus was 1–100 copies per well		
		The kit exhibited 98.8% sensitivity and 96% specificity		
		The strip exhibited 97.5% sensitivity and 96% specificity		

### 3.4 Nanoclusters for fungal diseases

Fungi have been implicated in infectious diseases. Diseases caused by fungal microorganisms are often difficult to diagnose precisely in terms of the specific species responsible and prove resistant to eradication. They also exhibit multidrug resistance to conventional treatments. Some fungal pathogens are associated with life-threatening systemic diseases in immunocompromised individuals.^115^ Therefore, the search for more potent alternatives is important.

Numerous reports have demonstrated the application of nanomaterials for the effective diagnosis and treatment of fungal diseases; however, nanoclusters (NCs) have shown superiority owing to the unique characteristics of these nanomaterials and their distinctive reactive oxygen species (ROS) production capacity.^116,117^ Many studies have reported that resistance to conventional fluconazole treatment often presents a limitation to successful treatment of *Candida albicans*. A study by Prateeksha *et al.* explored the development of eco-friendly silver nanoclusters using an extract from *Usnea longissima*, a symbiotic organism of algae and fungi. These nanoclusters were effective in suppressing the growth of fluconazole-resistant *Candida albicans*. This approach was developed as an alternative to chemically synthesized silver nanoparticles (NPs), which are considered toxic and generally not safe. *Usnea longissima* (UL) is known for its high bioflavonoid content, which contributes to its reducing properties, making it suitable as a template for silver nanocluster/nanoparticle production. Small (2–4 nm) silver nanoclusters (NCs) were synthesized by biotransforming UL polyphenolic metabolites into *O*-quinones through the addition of polyphenol oxidase.^118^ These silver NCs exhibited a sharp redox current, high surface-area-to-volume ratio, and significant pathogen-killing potential *via* oxidative stress.^117^ The fungicidal effect of silver NCs against *Candida albicans* was evaluated using clonogenic survival and disc diffusion assays. The results showed a significantly larger zone of inhibition than that of silver NPs and pure fluconazole. Silver NCs also demonstrated a higher rate of cell death than silver NPs and fluconazole. Further investigation revealed that *Candida albicans* was unable to attach to surfaces coated with silver NCs, while uncoated surfaces allowed easy attachment.^118^ One proposed mechanism for the fungicidal effects of silver NCs is linked to the reduction in oxygen consumption rate and activity level in the mitochondrial respiratory chain, as well as changes in the glycolysis status of *Candida* cells over time.^119^ In summary, the research found that silver NCs induced apoptosis in fluconazole-resistant *Candida albicans* by targeting reactive oxygen species (ROS)-dependent Ras signaling, potentially enhancing the efficacy of conventional antifungal agents.^118^ One proposed mechanism for the fungicidal effects of silver NCs is linked to the reduction in oxygen consumption rate and activity level in the mitochondrial respiratory chain, as well as changes in the glycolysis status of *Candida* cells over time.^119^ From a clinical perspective, these non-toxic, chemically free silver NCs may improve the prognosis of life-threatening fungal infections caused by fluconazole-resistant *Candida albicans* in patients.

The rise in clinical cases of fungal pathogens exhibiting multidrug resistance also prompted the development of a new formulation using the green synthesis of argirium silver nanoclusters (ASNCs). This innovation targets *Aspergillus niger*, a fungus that produces mycotoxins harmful to humans. Invasive aspergillosis (IA) can occur in immunocompromised patients undergoing transplantation or chemotherapy.^120^ The synthesized ASNCs were less than 2 nm in size and featured a remarkably stable structure with an Ag^0^ core surrounded by a shell of Ag^+^, Ag^2+^, and Ag^3+^, exhibiting exceptional redox properties. These nanoclusters demonstrated potent antifungal activity against both mycelia and spores, with a minimum inhibitory concentration (MIC) of 1.25 ppm after a week-long incubation at 28 °C. The proposed mechanism of action involves depolarization of the fungal cell membrane, leading to cell death. This was verified by observing changes in membrane properties in the presence of ASNCs using a fluorescent dye, which penetrated the cell upon membrane depolarization, bound to membrane proteins, and emitted intense green fluorescence (Fig. 7).^121^ The uniqueness of these ASNCs comes from the stable existence of Ag^2+^ and Ag^3+^ cationic species within a coordinated Ag_3_O_4_ lattice, in contrast to other AgNPs that contain only Ag^0^ and Ag^+^. The presence of Ag^2+^ and Ag^3+^ results in membrane depolarization and subsequent loss of function, causing fungal death, as Ag^0^ and Ag^+^ alone cannot depolarize cell membranes. Additionally, preclinical toxicity studies have revealed higher toxicity in fungal cells than in human cells.^121^ This innovative approach could significantly contribute to overcoming multidrug resistance in fungal therapy, potentially reducing the incidence of fungal-based infectious diseases. However, the study was limited to *in vitro* testing on a single strain and did not evaluate performance in complex environmental matrices.

**Fig. 7:**
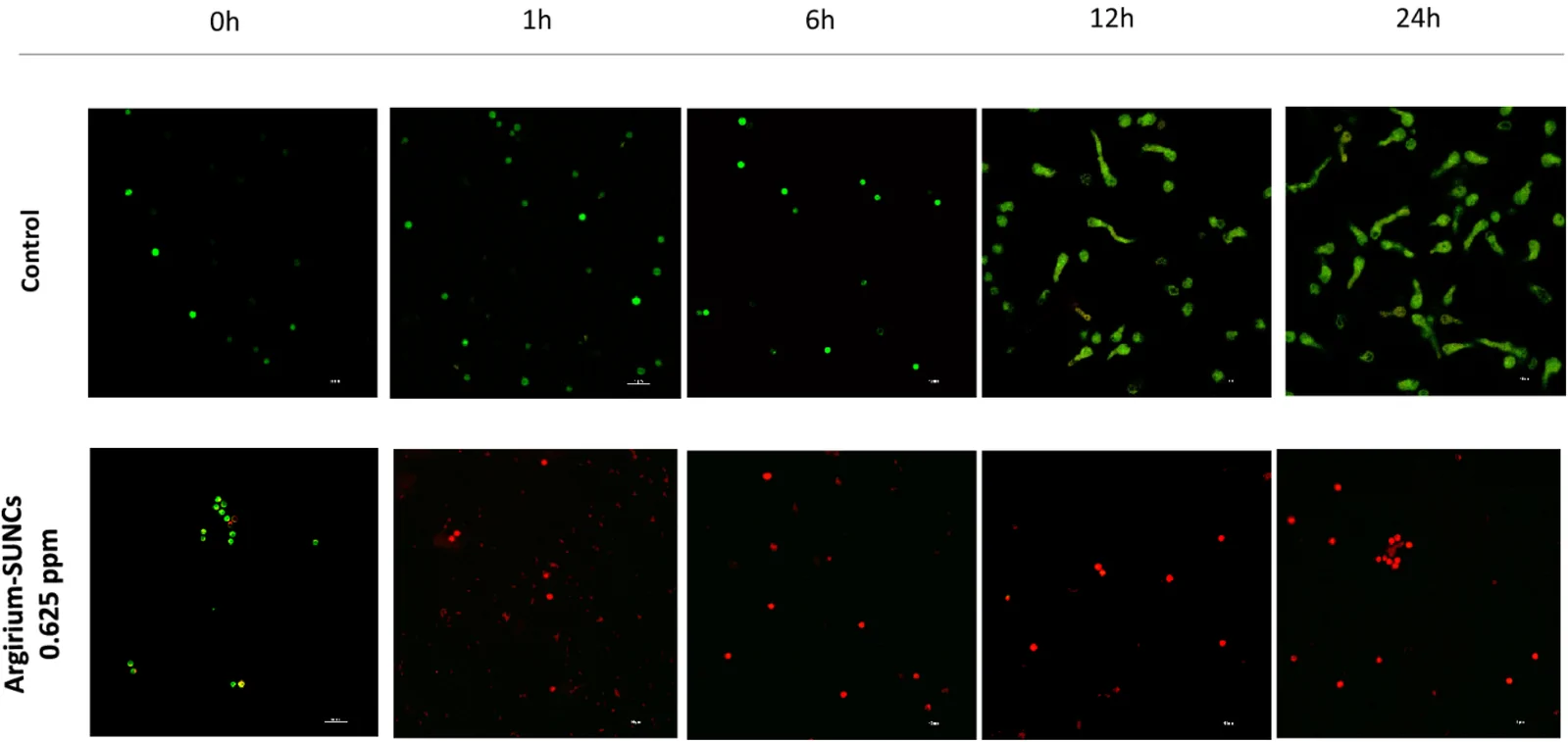
Fluorescence microscopy of fungal cells showing the effect of argirium silver nanoclusters on *A. niger* GM31 cell viability. Green stain indicates live and dead cells (intact fungal cells) while red indicates damaged fungal structure. Scale bar 20 µm.^121^ Reproduced from ref. 121 under a Creative Commons Attribution 4.0 International License.

A recent investigation explored the potential antifungal properties of TiO_2_ modified with small Ag and CuO nanoclusters in a core–shell configuration against *Aspergillus melleus* and *Penicillium chrysogenum*. TiO_2_ has long been recognized for its photoactivity, cost-effectiveness, and stability. However, its photoactivity is limited by UV irradiation, which restricts indoor applications.^122^ To expand TiO_2_'s functionality into the visible light spectrum and enable its use with indoor light sources, surface modification using plasmonic nanoparticles or nanoclusters, such as Au, Ag, and Cu, presents a promising avenue. Méndez-Medrano *et al.* enhanced TiO_2_ with silver and copper (Ag@Cu/TiO_2_) in a core–shell structure, experimenting with various Ag–Cu ratios (1 : 1, 1 : 3, 3 : 1). The 1 : 3 ratio, consisting of an Ag core and CuO shell deposited on TiO_2_, demonstrated the highest antifungal activity under both dark and visible-light conditions, with better efficacy observed in darkness. This antifungal effect was attributed to the adsorption of Cu nanoclusters, present in higher quantities, onto the Ag nanomaterials, which accelerated attachment and penetration into fungal cells. The nanoscale dimensions of the nanocluster system reportedly provide a larger surface area, facilitating the release of Ag and Cu ions and thereby inhibiting fungal growth.^122^

Rapid and accurate diagnosis is crucial for effective treatment of fungal infections, and nanoclusters (NCs) offer a promising solution. Several NC platforms have been explored for the detection of fungal pathogens. In many clinical scenarios, identifying the specific fungal species that cause infections is essential for appropriate treatment. For example, various *Candida* species show different susceptibilities to antifungal agents.^123^ Consequently, species identification may be necessary before antifungal therapy is initiated. Currently, the standard method for distinguishing between *Candida* species involves microbial culture and morphological observation, which can be challenging because of their similar appearances. Although molecular techniques such as rDNA analysis, phospholipid pattern differences, and cell wall protein identification have been developed, they are time-consuming and require specialized expertise.^124^

Fluorescent metal NCs can interact with fungal cell wall components, producing detectable signals that enable rapid and simple differentiation between species of a particular fungal pathogen. Cong *et al.* developed gold nanoclusters (AuNCs) decorated with 11-mercaptoundecanoic acid (MUA) and oligo(ethylene glycol) (OEG), allowing interfacial interactions between the ligands and *Candida* species' cell walls. The varying cell wall compositions of different *Candida* species resulted in distinct levels of association with AuNCs, leading to distinguishable fluorescence signals. Increased emission intensities were observed when *Candida albicans* and *Candida parapsilosis* were added to either AuNCs-MUA or AuNCs-MUA/OEG. With AuNCs-MUA, *Candida parapsilosis* exhibited a higher fluorescence signal than *Candida albicans*. Conversely, when AuNCs-MUA/OEG was introduced, the emission intensity was significantly higher for *Candida albicans* than for *Candida parapsilosis*. These variations in fluorescence emission confirmed the interaction between the surface ligands and the *Candida* cell wall. The two *Candida* species had different cell wall structures and compositions. *Candida albicans* possesses long, dense oligosaccharides in mannan, which mask hydrophobic proteins, whereas *Candida parapsilosis* has short oligosaccharides, exposing hydrophobic proteins.^125^ Due to these structural differences, *Candida parapsilosis* showed a higher fluorescence signal after incubation with AuNCs-MUA, attributed to hydrophobic interactions between AuNCs-MUA and the more hydrophobic surface of *Candida parapsilosis*. In contrast, following incubation with AuNCs-MUA/OEG, *Candida albicans* displayed a much stronger fluorescence signal resulting from hydrophilic interactions between AuNCs-MUA/OEG and the hydrophilic surface of *Candida albicans*.^126^ Without fluorescence, the two species are morphologically similar; however, NCs provide a strategy to easily distinguish them using ligand-functionalized AuNCs by leveraging hydrogen bonding and hydrophobic interactions at the biointerface.

Researchers have also demonstrated the application of DNA-templated silver nanoclusters (AgNCs) for the detection of aflatoxin B1 (AFB1), a highly carcinogenic compound produced by certain fungi. Aflatoxins, which are extremely toxic mycotoxins generated by fungal species such as *Fusarium*, *Aspergillus*, and *Penicillium*, pose serious public health concerns. AFB1 is particularly notorious for causing liver damage and various cancers. To address these health risks, Dadmehr *et al.* developed a detection method that integrated fluorescence and colorimetric analysis into a single platform. This approach utilizes two probes: one to enable aptamer strand hybridization and another to provide the necessary secondary structure. When AFB1 was present, a strong interaction between AFB1 and the aptamer occurred, resulting in aptamer dissociation. The liberated aptamer strand was hybridized with the second probe. Upon exposure to the ExoIII enzyme, a cleavage process occurred that released a substrate (heterostructure including a G-quadruplex) for AgNC synthesis, while the remaining portion engaged in further hybridization with free aptamers (Fig. 8). The resulting G-quadruplex enhanced the fluorescence activity and promoted the catalytic activity of AgNCs on 3,5,3′,5′-tetramethylbenzidine (TMB) in the presence of hydrogen peroxide (H_2_O_2_), enabling colorimetric detection of AFB1.^127^ This innovative technique, which combines fluorescence and colorimetric analysis, provides a straightforward, efficient, sensitive, and simple method for swift AFB1 detection in food samples.

**Fig. 8:**
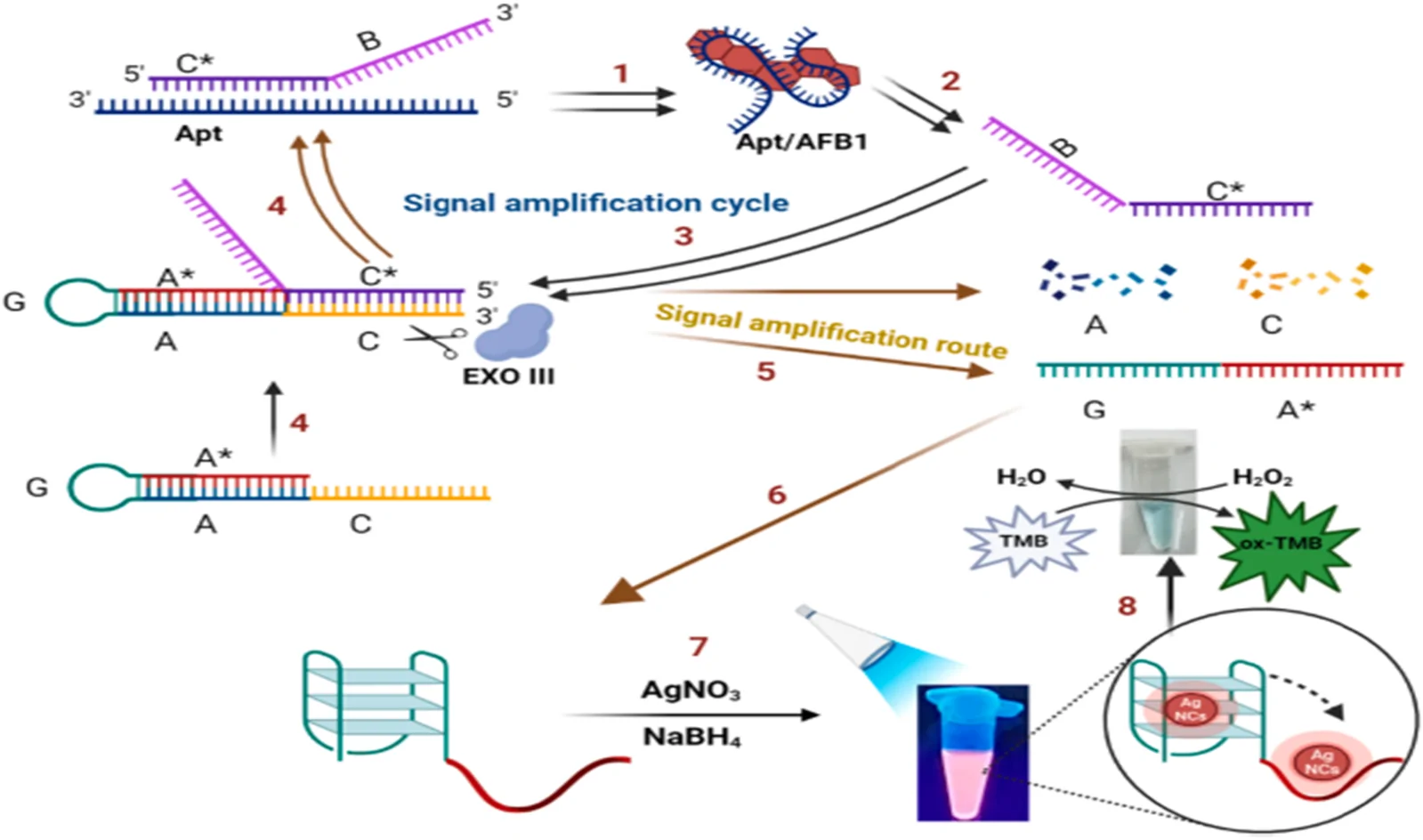
Mechanism for the detection of aflatoxin B1 using DNA template silver nanoclusters. NCs for fungal infections. Reproduced from ref. 127 with permission from Elsevier.

In conclusion, it is important to highlight that innovative metal nanoclusters can be synthesized using various substrates, including DNA and proteins. These nanoclusters show promise as diagnostic tools and antimicrobial agents to combat infectious fungal diseases (Table 4).

**Table 4:** Recent studies on the use of nanoclusters in the diagnosis and treatment of fungal infections

Composition	Method of synthesis	Target fungal species	Mechanism of action	Key finding	References
Gold nanoclusters (AuNCs)	Chemical reduction using Au(iii) and tetrakishydroxymethylphosphonium chloride, followed by ligand exchange (amide coupling for OEG modification)	*C. albicans*, *C. parapsilosis*	Interaction between AuNC surface ligands and fungal cell walls; hydrogen bonding and hydrophobic interactions depending on cell wall composition; induces distinct fluorescence enhancement for species identification	Enables rapid, label-free discrimination between *C. albicans* and *C. parapsilosis via* distinct fluorescence signals; interaction strength and fluorescence change depend on cell wall and ligand chemistry, not fungicidal but diagnostic	128
Biosilver nanoclusters (rsAgNCs, ∼2–4 nm)	Green synthesis using aqueous extract of usnioid lichen (*Usnea longissima*) and polyphenol oxidase-mediated reduction	Fluconazole-resistant *C. albicans* (NBC099)	ROS accumulation, mitochondrial potential loss, apoptosis, and Ras-cAMP signaling alter metabolism and activate cell death pathways	MIC 10 µg mL^−1^; strong fungicidal activity, apoptotic death, proteomic changes, non-toxic to mammalian cells	129
Gold–silver nanoalloys with triple helix glucan (lentinan, LNT)	Microwave-assisted reduction with LNT (triple-helix β-glucan)	*C. albicans*	Silver ions disrupt biofilm and cause fungal cell membrane damage; encapsulation in LNT improves biocompatibility	MIC (Ag content): 8–32 µg mL^−1^ (best at Ag : Au = 5 : 1); strong dose-dependent antibiofilm action; no cytotoxicity to mammalian cell lines	130
Blue-emitting gold nanoclusters	Green synthesis using *Argyreia nervosa* leaf extract	Fungicide (hexaconazole) detection; bioimaging with *S. cerevisiae*	Hexaconazole binding causes aggregation and strong (<80%) fluorescence quenching of AuNCs; probe internalizes and emits in yeast cells	Highly sensitive fluorescence “turn-off” probe for hexaconazole fungicide (LOD: 21.94 nM, linear range: 0.025–180 µM); selective *vs.* other pesticides; excellent bioimaging and non-toxic to mammalian cells	131
TiO_2_ modified with core–shell AgCuO nanoclusters	Radiolytic reduction of Ag/Cu salts on TiO_2_–P25	*A. melleus*, *P. chrysogenum*	Synergistic Ag/CuO effects: membrane disruption, ROS generation, ion leaching, growth inhibition, and mycotoxin suppression	Ag : CuO 1 : 3 ratio offered highest inhibition (in dark and visible light), prevents sporulation and possible mycotoxin droplets, best for indoor surface coatings, highly effective at decreasing colony diameter	132
Riboflavin-protected ultrasmall silver nanoclusters	Chemical reduction of AgNO_3_ by riboflavin	*S. aureus*, *E. coli*, and *C. albicans*	Disruption of bacterial cell membrane integrity, induction of intracellular ROS, penetration, and aggregation inside cells	RFAgNCs displayed superior bactericidal and fungicidal effects compared to RF, AgNPs, or mixed RF + AgNPs; minimal toxicity to red blood and mammalian cells; promising use for biomedical, water sterilization, and textiles	82

## 4 Benefits and challenges of nanoclusters in the diagnosis and treatment of infectious diseases

Metal NCs exhibit many beneficial properties for application in the management of infectious diseases. Gold, silver, copper, and alloy NCs have been explored for diagnosing and treating infectious diseases, including those caused by multidrug-resistant organisms. The manipulation and tailoring of metal nanoclusters at the atomic level enable them to act as theranostic agents for infections.^43^ Nanoclusters have shown great potential as luminescent emitters with good quantum yield and better biocompatibility than traditional emitters. They can be employed for rapid detection of microorganisms as well as for treating infections. For instance, Wang *et al.* synthesize Au NCs using an antimicrobial peptide and glutathione as the ligands. The nanocluster exhibited both aggregation-induced emission and enhanced antibacterial effects.^43^ Similarly, DNA–Ag nanoclusters showed promising results for the facile detection of SARS-CoV-2, HIV and influenza virus.^133^ In addition, nanoclusters can be used in antimicrobial photodynamic therapy, where luminescent emitters generate ROS to kill microbes.^43^

Metal NCs exhibit many properties that directly or indirectly aid in disrupting cell walls and membranes, destroying intracellular components, generating reactive oxygen species, releasing metal ions, and the uptake of antimicrobial agents. For instance, due to their ultrasmall sizes, metal NCs can quickly internalise into microbial cells *via* the cell wall pores. This promotes ROS generation to oxidise cell membranes and disrupt microbial metabolism, enhancing their antimicrobial activity.^134^ Also, NCs provide excellent catalytic action due to their several active sites and thus generate higher levels of ROS, improving their antimicrobial potency. Interestingly, metal nanoclusters are traceable due to their excellent photoluminescence activity.^135^ This feature can be utilised for real-time tracking of their activities in biological systems and in studying their mechanisms of action.^136^ In addition, the precise atomic structure of nanoclusters enables a better understanding of their structure–activity relationships as antimicrobial agents. In addition, metal NCs have excellent pharmacokinetics, biodegradation characteristics, and renal clearance, which are essential for clinical translation.

Metal NCs can be incorporated into other bioactive materials, such as polymers, upconversion nanoparticles and 2D nanomaterials, to obtain enhanced antimicrobial effects.^137,138^ They can also be combined with another antimicrobial agent with a different mechanism of action to achieve synergistic antimicrobial activity. For instance, an antimicrobial peptide, daptomycin, conjugated with gold nanocluster, gave a synergistic antimicrobial effect and superior bactericidal activity against multidrug-resistant bacteria.^139^

Nanoclusters have been widely explored for targeting biofilm, a barrier to antibiotic penetration and activation and a major cause of antimicrobial resistance. Unlike traditional antimicrobial agents, which show poor penetration and weak antimicrobial activity in the acidic biofilm microenvironment, nanoclusters have been reported to have excellent antimicrobial activity against biofilms. Wu *et al.*^140^ reported the activity of Au NCs against *Shigella* biofilm. The NCs destroyed the bacterial cell membrane and acted on the genes involved in oxidative metabolism to increase ROS levels, thereby killing the *Shigella* cells. Also, silver nanoclusters with pH-responsive ligands showed enhanced bactericidal effect in bacterial biofilm.^141^ The system was designed to exhibit charge reversal and disassembly of the nanoclusters upon reaching the acidic environment. This resulted in rapid leaching and enhanced local accumulation, retention, and activity of silver ions. Superior *in vitro* and *in vivo* activity of silver nanocluster in multi-drug-resistant biofilm-induced severe pyomyositis mouse model was also reported. Besides environment-responsive nanoclusters, ligands have been used to target nanoclusters to infection sites. For instance, conjugating d-mannose to bovine serum albumin-stabilized gold nanoclusters helped to target them to bacterial cells.^142^ Following their uptake, the entrapped drug was released inside the bacteria, causing reactive oxygen species production and membrane permeability alteration, ultimately resulting in bacterial inhibition.

From these advantages, ultrasmall NCs are promising, new-generation nanoantibiotics for combating infections. Despite the enhanced antimicrobial efficacy, there are stability and safety concerns on nanoclusters for their clinical application. Many factors are implicated in the stability and toxicity of nanoclusters, including their size, composition and surface functionalities. Due to their ultrasmall size and unsaturated coordination, nanoclusters are chemically reactive and prone to aggregation and oxidation reactions. They also suffer from long-term stability, hindering their clinical applications, especially in devices. The physicochemical properties of the metals and ligands influence their toxicity. Unlike l-glutathione-based nanoclusters, d-glutathione-capped cationic gold nanoclusters do not exhibit cellular cytotoxicity, hemolysis, or acute organ damage.^143^ The surface charge of the nanocluster is another critical factor in determining the biodistribution and toxicity of the nanosystem, as negatively charged nanoclusters were found to exhibit lower excretion rates and higher cellular uptake than positively charged gold nanoclusters.^144^ Optimizing these variables can help increase the safety profile of nanoclusters.

Another problem with nanoclusters in infectious disease diagnosis is their poor fluorescence intensity. Most nanoclusters have a weaker fluorescence (quantum yield < 10%) compared to 95–100% quantum yield from quantum dots and organic dyes. Templating Ag, Au, or Cu with DNA or 3-aminophenyl boronic acid is one of the strategies investigated to enhance their fluorescence intensity.^145,146^

Despite these challenges, metal nanoclusters hold great promise in medicine. Researchers are devising strategies to overcome their poor stability and toxicity. Silver and copper nanoclusters show less stability and biocompatibility but exhibit better antibacterial activity than gold nanoclusters.^147,148^ Hence, many strategies are explored to increase the antimicrobial efficacy of gold-based nanoclusters and improve the stability of Ag and Cu nanoclusters. Such strategies include modifying their physicochemical properties, alloying, and incorporating other anti-infective agents. For example, hybrid nanomaterials containing these nanoclusters are being explored in treating and diagnosing infectious diseases. Also, metal alloy NCs, such as AuAg NCs and AuPt NCs, which are more stable than single metal nanoclusters, are being investigated. However, the effect of alloying on the antimicrobial activity should be adequately considered, as Zheng *et al.* reported a reduction in the antibacterial activity of gold–silver alloyed nanoclusters.^61^

## 5 Limitations to clinical translation of nanoclusters

Despite the remarkable progress achieved with nanoclusters in experimental research, their movement into clinical practice remains hindered by numerous challenges. One of such limitations is the challenge of scaling production reproducibly. This is especially difficult because the atomic precision that defines nanoclusters, including their well-controlled structures and ligand shells, makes them very sensitive to small changes in synthesis conditions. Although laboratory protocols can reliably generate small batches, attempts to scale production to levels required for preclinical or clinical use often result in variability in size distribution, surface chemistry, as well as biological properties. For instance, several studies have reported that differences in precursor quality, temperature control, or ligand concentration during synthesis can alter important properties, making it difficult to guarantee uniformity across batches.^7,12,15,16^ Furthermore, the issue of stability adds another layer of complexity, as many nanoclusters are prone to aggregation or ligand exchange when exposed to changes in pH, ionic strength, or light during storage and transport. As such, these factors complicate the process of developing GMP compliant large-scale production methods for nanoclusters.

In addition, there have been concerns regarding the safety of these systems. Several studies have reported that nanoclusters are biocompatible and that their small size makes them ideal for renal clearance; however, evidence suggests that clearance profiles differ significantly depending on the choice of ligand and surface modifications. For instance, glutathione-stabilized gold nanoclusters are often cleared rapidly through the kidneys. At the same time, protein-coated clusters show a greater tendency to accumulate in the liver and spleen, raising questions about long-term retention and potential toxicity.^149^ Their capacity to generate reactive oxygen species supports their antimicrobial activity, but uncontrolled ROS can also damage host tissues. The immune response is another area of uncertainty, as changes in surface chemistry can trigger cytokine release or inflammatory reactions. It is therefore pertinent to look into obtaining comprehensive toxicological data covering both short-term and chronic exposures across multiple species to provide a better understanding of the safety profile of these systems.

Another challenge to the clinical translation of these systems is the limited data to demonstrate significant efficacy in complex biological systems. Many studies use simplified models of infection, such as single-strain bacterial cultures or basic animal systems, that do not reflect the diversity of clinical diseases. Some reports have tried to look at the penetration of biofilms *in vitro* as a strategy to depict *in vivo* infections; however, this does not always translate into reliable outcomes *in vivo*. Similarly, intracellular pathogens that persist in phagosomes or protected tissue sites remain difficult to reach at effective concentrations. Although nanoclusters can act through several antimicrobial mechanisms, including oxidative stress, membrane disruption, and enzyme inhibition, their effects against intracellular pathogens that persist in phagosomes have not been extensively studied. These microorganisms are especially important as they play a vital role in the development of resistance. Also, because the pharmacokinetics and pharmacodynamics of these systems are not yet fully characterized, there are critical gaps in knowledge linking nanocluster composition, size, ligand identity, and surface charge with biodistribution, clearance, and therapeutic window, making rational dosing strategies difficult to establish.

The processes involved in regulatory approvals further complicate the clinical translation of nanoclusters. This is mainly because most frameworks for drug approval were designed for small molecules or biologics, and not atomically unique nanomaterials like the nanoclusters. Because nanoclusters possess sizes that fall between these categories, they exhibit molecular-like properties alongside nanoscale behaviors, which further complicates their characterization. Regulatory bodies usually require comprehensive characterization, including data on composition, ligand structure, stability, and batch consistency, details that are lacking in many published studies. Many studies utilize standard toxicity tests designed for conventional drugs, which may fail to capture the unique interactions of nanomaterials with biological systems. These factors have contributed to the absence of a robust regulatory pathway for nanoclusters, which in turn discourages industrial investment. Differences in guidelines across jurisdictions add to the uncertainty, making it difficult to design development programs that will be acceptable to multiple agencies.

These challenges point to the importance of collaborative strategies in order to bring about the clinical translation of these systems. More attention should be paid to standardizing characterization and reporting practices. This would make results more comparable across studies and provide regulators with the evidence needed for evaluation. Furthermore, developing robust, reproducible, as well as scalable methods of synthesis that also comply with Good Manufacturing Practice is essential to bridge the gap between the laboratory and the clinic. Preclinical studies should employ standardized infection models that reflect the complexity of clinical disease, including biofilm-associated infections and intracellular pathogens. In addition, it would also be very beneficial to engage proactively with regulators, especially during the early stages of development, which will help clarify requirements for approval and help align experimental outcomes with clinical needs.

In summary, nanoclusters hold considerable promise as tools for diagnosing and treating infectious diseases, but significant barriers hinder their translation into clinical practice, including production, safety, efficacy, and regulatory issues. These barriers, though tough, are not insurmountable but require an interdisciplinary collaboration involving clinicians, regulators, ethicists, and public health experts. It is only through a transparent and coordinated approach that nanoclusters can progress from experimental constructs to reliable tools for next-generation infectious disease management.

## 6 Conclusion and future perspectives

This review provides new insights into nanoclusters and their use in diagnosing and treating infectious diseases. Due to their ultrasmall size and large surface area, nanoclusters have chemical, physical and electrical properties different from larger nanoparticles. Several studies have reported enhanced antimicrobial activity using these nanostructures. Their efficacy against multidrug-resistant microbes and microbial biofilms and their use as tracers are of great interest. In addition, capping them with targeting ligands such as DNA, proteins, and peptides has yielded promising results.

In spite of the many benefits, the translation of nanoclusters into clinical use is hindered by many obstacles. The biodistribution and interaction of nanoclusters with biological systems have yet to be fully understood. Fewer studies are reported on the use of these nanostructures in viral and fungal diseases. More research is needed in this area to fully understand their mechanisms of antimicrobial action and facilitate rational drug design. The size and charge of nanoclusters have been found to play a significant role in their activities as they influence their penetration into microbial cells and the generation of oxidative stress. The enzymatic activity (peroxidase and oxidase-like activities) of these nanoantibiotics, vital for their antimicrobial efficacy, is also affected by size.^150^ Moreover, the composition and size of nanoclusters have been implicated in the aggregation-induced emission effect of metal nanoclusters. Hence, the impact of the type and properties of the core materials and ligands utilised in the synthesis of antimicrobial nanoclusters on their performance should be elucidated.

Nanoclusters exhibit inherent chirality and structural isomerisation due to the arrangement of metallic core and protective ligands. These features enhance their diversity and biomedical applications.^151^ However, their effects on intracellular uptake, biodistribution, and toxicity should be given proper consideration when designing nanoclusters. Furthermore, ligands interact with biological components in blood, such as proteins, leading to biomolecular corona formation. The implication of this phenomenon in the efficacy of nanoantibiotics should be characterised. Likewise, the interactions of NCs with intracellular barriers, such as endocytosis, lysosomal degradation, rigid plasma membrane and efflux pumps, should be investigated. A better understanding of the relationships between the antimicrobial activity and the properties of NCs will facilitate the rational design and development of nanoclusters as an effective therapy for infectious diseases.

Future research should focus on developing biomolecule-stabilised metal nanoclusters as sensors for diagnosing COVID-19 and other viral infections. Protein-stabilised nanoclusters have been reported to be better than conventional fluorescence probes based on fluorescence lifetime, biocompatibility and stability. Many nanoclusters, especially the non-noble metal and non-metal based nanostructures, have low quantum yield compared to quantum dots and other commonly used fluorophores. More effort should be geared towards developing efficient strategies to improve their quantum yield. Alloying is a widely explored strategy for enhancing the photoluminescence efficacy as well as stability of nanoclusters. However, there is a need to understand the effect of alloying on the pharmacological action, especially the antimicrobial activity of nanoclusters. Hybrid nanomaterials have been reported to significantly boost the photoluminescence efficacy, stability, catalytic actions and antimicrobial efficacy of nanoclusters. There is a need to explore the use of different atomicity, metals or ligands in generating multifunctional hybrid nanoclusters. A joint effort of material scientists, nanobiotechnologists, pharmaceutical scientists and clinicians is needed to move these nanomaterials into the clinics.

## Conflicts of interest

The authors declare no competing interests.

## Data Availability

No primary research results, software or code have been included and no new data were generated or analysed as part of this review.
